# Pharmacologic Inhibition of SHP2 Blocks Both PI3K and MEK Signaling in Low-epiregulin HNSCC via GAB1

**DOI:** 10.1158/2767-9764.CRC-21-0137

**Published:** 2022-09-26

**Authors:** Richard Kurupi, Konstantinos V. Floros, Sheeba Jacob, Ayesha T. Chawla, Jinyang Cai, Bin Hu, Madhavi Puchalapalli, Colin M. Coon, Rishabh Khatri, Giovanna Stein Crowther, Regina K. Egan, Ellen Murchie, Patricia Greninger, Krista M. Dalton, Maninderjit S. Ghotra, Sosipatros A. Boikos, Jennifer E. Koblinski, Hisashi Harada, Yue Sun, Iain M. Morgan, Devraj Basu, Mikhail G. Dozmorov, Cyril H. Benes, Anthony C. Faber

**Affiliations:** 1 VCU Philips Institute, School of Dentistry and Massey Cancer Center, Richmond, Virginia.; 2 Department of Pathology, Virginia Commonwealth University School of Medicine, Richmond, Virginia.; 3 Massachusetts General Hospital Cancer Center, Boston, Massachusetts.; 4 Department of Medicine, Harvard Medical School, Boston, Massachusetts.; 5 Georgetown Lombardi Comprehensive Cancer Center, Washington, District of Columbia.; 6 Department of Otorhinolaryngology–Head and Neck Surgery, The University of Pennsylvania, Philadelphia, Pennsylvania.; 7 Department of Biostatistics, Virginia Commonwealth University School of Medicine, Richmond, Virginia.; 8 Department of Pathology, Virginia Commonwealth University, Richmond, Virginia.

## Abstract

Preclinical and clinical studies have evidenced that effective targeted therapy treatment designed against receptor tyrosine kinases (RTKs) in different solid tumor paradigms is predicated on simultaneous inhibition of both the PI3K and MEK intracellular signaling pathways. Indeed, reactivation of either pathway results in resistance to these therapies. Recently, oncogenic phosphatase SHP2 inhibitors have been developed with some now reaching clinical trials. To expand on possible indications for SHP099, we screened over 800 cancer cell lines covering over 25 subsets of cancer. We found head and neck squamous cell carcinoma (HNSCC) was the most sensitive adult subtype of cancer to SHP099. We found that, in addition to the MEK pathway, SHP2 inhibition blocks the PI3K pathway in sensitive HNSCCs, resulting in downregulation of mTORC signaling and antitumor effects across several HNSCC mouse models, including an human papillomavirus (HPV+) patient-derived xenograft. Importantly, we found low levels of the RTK ligand epiregulin identified HNSCCs that were sensitive to SHP2 inhibitor, and, adding exogenous epiregulin mitigated SHP099 efficacy. Mechanistically, epiregulin maintained SHP2–GAB1 complexes in the presence of SHP2 inhibition, preventing downregulation of the MEK and PI3K pathways. In the presence of SHP2 inhibitor, HNSCCs are highly dependent on GAB1 for their survival and knockdown of GAB1 is sufficient to block the ability of epiregulin to rescue MEK and PI3K signaling. These data connect the sensitivity of HNSCC to SHP2 inhibitors and to a broad reliance on GAB1-SHP2, revealing an important and druggable signaling axis. Overall, SHP2 inhibitors are being heavily developed and may have activity in HNSCCs, and in particular those with low levels of epiregulin.

**Significance:**

This work identifies a novel role of SHP2 inhibitor by dual downregulation of PI3K and MEK pathways, through loss of GAB1 activation and disruption of GAB1 complexes in low-epiregulin HNSCC.

## Introduction

Head and neck squamous cell carcinoma (HNSCC) is the eighth most common cancer in men in the United States and is significantly more prevalent in the rest of the world (1). There are roughly 50,000 new cases per year and 10,000 deaths annually in the Unites States (2). A total of 60% of these cancers arise from oral sites, which include the oral cavity and the oropharynx. Oral HNSCC has undergone a rapid shift in epidemiology: oropharyngeal cases are now usually human papillomavirus (HPV) related (3), and HPV(+) disease is predicted to surpass HPV(−) HNSCC in incidence by 2030. Locoregional disease for HNSCC is treated with surgery, high-dose radiation, cisplatin, and/or mAbs targeting the EGFR. Despite aggressive and toxic multimodality treatment, more than half of all advanced stage HNSCCs have lethal outcomes, and a significant number of HPV+ HNSCCs recur (4). For inoperable, relapsed-refractory cases, and those with distant metastasis at presentation, available therapies are limited to platinum-based palliative therapy, the anti-EGFR antibody cetuximab, and with immunotherapy. Anti-PD1 agents demonstrate a modest 15%–20% response rate (5, 6). Therapy beyond these agents remains exploratory (7), leaving a large number of patients with HNSCC with advanced disease with a high probability of death.

Cetuximab was shown to provide clinical benefit when added to radiation (8) and platinum-based palliative therapy (9) about a decade ago. However, despite widespread use of EGFR antibody, the survival benefit is minimal (10). Constitutive activation of the PI3K-mTORC and Ras-Raf-MEK-ERK oncogenic signaling pathways are frequent occurrences in solid malignancies and are often the result of mutational activation (e.g., *PIK3CA* and *RAS* mutants) or aberrant expression of upstream regulators [e.g., receptor tyrosine kinase (RTK) activating mutation or amplification]. Direct targeting of both these pathways with small-molecule inhibitors has elicited widespread efficacy across a number of different solid tumor paradigms (11–13) and RTK inhibitors that have proven successful clinically block both of these pathways. For instance, EGFR inhibitors block both the PI3K and MEK pathway in *EGFR*-mutant lung cancers, and resistance can occur when either of these pathways is reactivated (13). On the other hand, with the exception of *BRAF*-mutant melanoma (14), clinically targeting either the PI3K or MEK pathway alone has demonstrated minimal success, largely due to feedback activation of the unblocked pathway (13, 15, 16). Unfortunately, clinical trials implementing the combination of PI3K and MEK inhibitors revealed targeting both pathways directly and simultaneously are highly toxic. Therefore, successfully targeting both PI3K and MEK pathways simultaneously underlies effective targeted therapy treatment, but for this to be attainable, the cancer requires a druggable addiction usually in the form of an upstream RTK. Examples of these paradigms include EGFR inhibitors in *EGFR*-mutant non–small cell lung cancer (NSCLC) and ALK inhibitors in *ALK*-translocated NSCLC.

SHP2 is one of the two SH2 domain–containing protein tyrosine phosphatases and is encoded by the *PTPN11* gene. Binding sites for the SHP2 SH2 domains that promote activation of the phosphatase are found in RTKs like EGFR and scaffolding adaptors. SHP2 has long been considered an attractive drug target and SHP099 is a novel allosteric SHP2 inhibitor that presents with good selectivity for RTK-driven cancer models (17). Recently, we (18) and others (19) have demonstrated that SHP099 can also sensitize to other targeted therapies, through suppression of the MAPK/ERK pathway. GAB1 is an important scaffolding adaptor that when phospho-tyrosine activated, engages SHP2, bridging SHP2 with active RTKs to downstream PI3K (through the regulatory subunit p85) and RAS/MEK/ERK pathways, through still disputed ways (20, 21). Indeed, SHP2 hyperactivating mutants exerts at least some of if its transforming potential through increased binding to GAB1 (20, 22).

The Center for Molecular Therapeutics (CMT) is a high-throughput screen (HTS) screening platform which enables discoveries of new sensitivities of often unsuspecting drugs to genetically defined subsets of cancers (23). Herein, we performed an HTS of SHP099, a specific SHP2 inhibitor with limited off-target effects (24), across hundreds of tumor-derived cell lines and found that HNSCC models were among the most sensitive. Surprisingly, sensitivity of SHP099 did not correlate with sensitivity to MEK pathway inhibitors. Instead, SHP2 inhibition led to downregulation of both MEK/ERK and PI3K signaling through inhibiting GAB1, which we found to be a critical protein for HNSCC survival. The loss of both signaling pathways converges on the mTORC pathway, as commonly seen in solid tumors (25), and underlies the hypersensitivity of a subset of HNSCCs to SHP2 inhibition.

## Materials and Methods

### Cell Lines

The HNSCC cell lines, SCC-9, JHU-022, HSC-4, BHY, and BICR22 were procured from the CMT at Massachusetts General Hospital Cancer. SCC-9, JHU-022, HSC-4, BHY, and BICR22 cell lines were cultured in DMEM/F12 (Corning Cellgro) supplemented with 10% FBS (Gibco) and 1 μg/mL penicillin and streptomycin. These cell lines undergo regular short tandem repeat (STR) testing at CMT but were not STR tested at VCU following screening. Cell lines were passaged up to 10 times following thawing prior to experimentation. No testing for *Mycoplasma* was performed in the laboratory.

### High-throughput Drug Screen

The SHP099 screen was performed at the CMT at the Massachusetts General Hospital across authenticated cell lines from GDSC collection as described previously (26).

### Analysis of mRNA Expression

RNA expression of ErbB ligands was obtained and analyzed through the R2: Genomics Analysis and Visualization Platform (http://hgserver1.amc.nl) using the GDSC-based Celline Cancer Drug (Sanger) dataset (Array Express Accession: E-MTAB-3610).

### Cell Viability Assays

HNSCC cell lines were seeded at 2 × 10^3^ cells per well in a 96-well microtiter plate. Twenty-four hours after seeding, cells were treated with varied concentrations of SHP099 and RMC-4550 (0–10 μmol/L) for 7 days, followed by measurement of cell viability by CellTiter-Glo protocol (Promega). Alternately, HNSCC cells were seeded at 2 × 10^3^ cells/well in a 96-well microtiter plate. Twenty-four hours after seeding, cells were treated with fixed concentration of SHP099 (10 μmol/L), alpelisib (3 μmol/L), and trametinib (10 nmol/L) and combination for 3 days, followed by measurement of cell viability measurements as above. Percent viability was constrained to a maximum of 100.

### Crystal Violet Assays

HNSCC cells were seeded at 5 × 10^4^ cells per well in a 6-well dish and treated the following day with 1 and 5 μmol/L SHP099 or RMC-4550 as indicated. Cells were stained with 0.1% crystal violet (Sigma-Aldrich) when no-treatment control cells reached confluency, at the end of approximately 7–10 days.

### siRNA Knockdown Experiments

The GAB1#1 siRNA (catalog no. D-003553-03-0005), GAB1#2 siRNA (catalog no. D-003553-04-0005), and scramble siRNA (catalog no. D-001810-01-20) were purchased from Dharmacon. All siRNAs were transfected using Lipofectamine RNAi MAX Reagent (Invitrogen) according to manufacturer's instructions.

### Antibodies and Inhibitors

Primary antibodies used for Western blotting were as follows: p-Tyr (sc-508), GAPDH (sc-32233), SHP2 (sc-7384), MCL-1 (sc-819) from Santa Cruz Biotechnology; p-EGFR (1068) (3777), p-ErbB2 (1248) (2247), ErbB3 (4754), p-ErbB3 (1289) (4791), p-ErbB3 (1328) (14525), p-ErbB4 (1284) (4757), p-Akt (308) (4056), p-Akt (473) (4060), p-Erk (202/204) (4370), p-P70S6K (389) (9205), p-4EBP1 (37/46) (2855), BIM (2933), BCL-xL (2764), p-P90RSK (380) (9341), p-S6 (235/6) (4858), p-S6 (240/4) (5364), c-Myc (5605), p-SHP2 (542) (3751), GAB1 (3232), p-GAB1 (627) (3233), p-GAB1 (659) (12745), β-ACTIN (4970), from Cell Signaling Technology. Secondary antibodies used were mouse IgG (GE Healthcare Life Sciences; NXA931) and rabbit IgG (GE Healthcare Life Sciences; NA934). Alpelisib and trametinib were purchased from AbMole Biosciences. RMC-4550 was purchased from Selleckchem. The SHP2 inhibitor (SHP099) for cell culture and *in vivo* experiments was purchased from MedchemExpress and AbMole Biosciences and was dissolved in DMSO at a stock concentration of 10 mmol/L for *in vitro* experiments. Human recombinant growth factors epiregulin (EREG) was purchased from Sigma-Aldrich (SRP3033) and neuregulin-1 (5898-NR) was purchased from R&D Systems.

### Western Blotting

Cell lines, tumors from cell-line xenografts, and tumors from patient-derived xenografts (PDX) were prepared and lysed in lysis buffer (20 mmol/L Tris, 150 mmol/L NaCI, 1% NP-40, 1 mmol/L EDTA, 1 mmol/L EGTA, 10% glycerol, and protease and phosphatase inhibitors). The samples were incubated on ice for 30 minutes, then centrifuged at 14,000 rpm for 10 minutes at 4°C. Tumor lysates were homogenized with Tissuemiser (Thermo Fisher Scientific) in the lysis buffer described previously, incubated for 30 minutes on ice, and centrifuged at 14,000 rpm for 10 minutes at 4°C. Protein concentration was determined by BCA Protein Assay (Pierce). Proteins were resolved using the NuPAGE Novex Midi Gel system on 4% to 12% Bis–Tris gels (Invitrogen), transferred to polyvinylidene difluoride membranes (PerkinElmer) in transfer buffer (Bio-Rad) with 20% methanol. Following transferring, the membrane was blocked in PBS-T with 5% nonfat milk for 1 hour and then incubated with the indicated antibodies overnight. After secondary antibody (GE Healthcare) incubation, the antibodies on the membranes were detected with the Syngene G: Box camera (Synoptics). Representative blots are shown in the figures.

### Immunoprecipitation Assay

A total of 500 μg of lysates were incubated with p85 antibody (5,000 ng; EMD Millipore, catalog no. ABS233), or SHP2 antibody (5,000 ng; Santa Cruz Biotechnology, catalog no. sc-7384), or rabbit IgG antibody (5,000 ng; Santa Cruz Biotechnology, catalog no. sc-2027). Following the addition of 25 μL of 1:1 PBS:prewashed Protein A Sepharose CL-4B beads (catalog no. 17-0963- 03; GE Healthcare Life Sciences) to the antibody/lysate mix, samples were incubated with rotating motion overnight. Equal amounts of extracts (5% of immunoprecipitated protein) were prepared in parallel.

### Animal Studies

For the BHY and HSC-4 xenograft HNSCC models, 5 × 10^6^ cells were injected subcutaneously into the right flank of (6–8 weeks) male NOD/SCID gamma (NSG) mice in a 1:1 ratio of cells to Matrigel (Corning, 354248). Treatment began when tumors reached approximately 100 to 200 mm^3^, and mice were randomized into treatment cohorts, with 5–8 mice per cohort. Tumor size and mouse weight were measured approximately 3 days per week using a digital scale and calipers, where tumor volume was calculated as height × width × width × 0.52, with height the larger of the two measurements. SHP099 was administered via oral gavage once per day, 6 days per week. SHP099 was dissolved in 0.6% hydroxypropyl methycellulose, 0.4% Tween80, and 0.9% saline for a final dosage of 75 mg/kg of body weight. All mice were euthanized at the end of 30 days of treatment. The PDXs were previously established under University of Pennsylvania Institutional Review Board protocol #417200. They were implanted, passaged, and cryopreserved as described previously (27) using NOD/SCID/IL-2R γ^−/−^ (NSG) mice. All animal experiments were approved by the Virginia Commonwealth University Institutional Animal Care and Use Committee (protocol# AD10001048).

### DepMap Data Analysis

Gene codependency data were obtained from DepMap consortium (https://depmap.org/portal/), which included mutation data, as well as CRISPR and combined RNAi results published in Q3 of 2020. The relationship between CRISPR screen results and gene codependency was studied (https://doi.org/10.6084/m9.figshare.12931238.v1).

### Data Availability Statement

The data generated in this study are available within the article and its Supplementary Data.

## Results

### HNSCC are Hypersensitive to SHP2 Inhibition through Dual PI3K and MEK Inhibition

**FIGURE 1:**
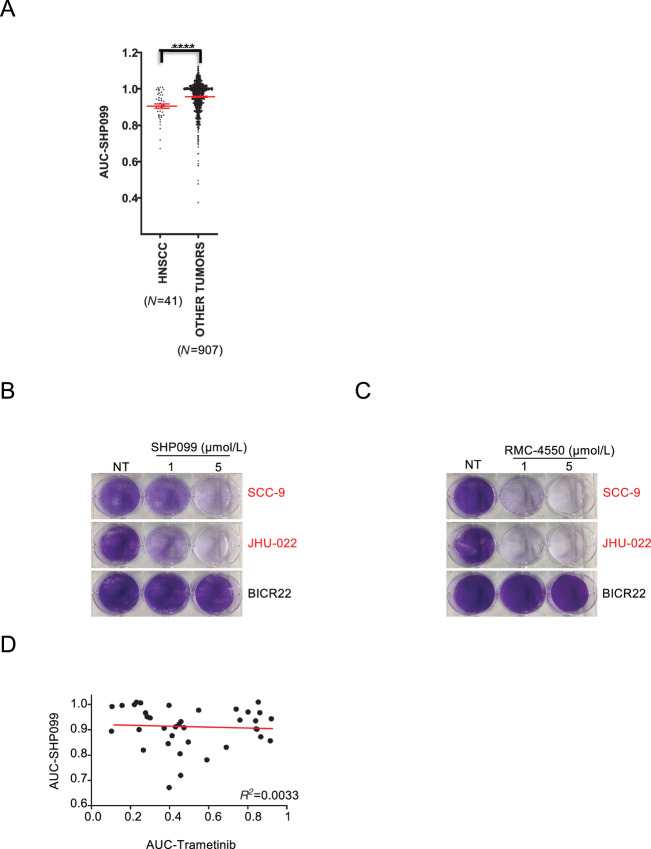
HNSCC cell lines are sensitive to the SHP2 inhibitor SHP099. **A,** Scatter plots showing SHP099 AUC in HNSCC cell lines (other tumors exclude neuroblastomas). Statistical significance was determined using the Mann–Whitney *U* test. ****, *P* < 0.0001. **B,** JHU-022, SCC-9, and BICR22 cell lines were treated with 1 or 5 μmol/L SHP099 and stained with crystal violet after 7 days. **C,** JHU-022, SCC-9, and BICR22 cell lines were treated with 1 and 5 μmol/L RMC-4550 and stained with crystal violet after 7 days. **D,** AUC for SHP099- and trametinib-treated cell lines were plotted to assess correlation in sensitivities. Correlation analysis was performed using Spearman correlation, *P* = not significant. NT = no treatment.

SHP099 is the first specific SHP2 inhibitor and a structurally related SHP2 inhibitor, TNO155, is now in clinical trials (NCT03114319). The SHP099 effect on cancer cells has been the subject of various recent focused studies (17, 19). Here, we evaluated whether a comprehensive drug screening of SHP099 on our HTS platform would reveal an unsuspected indication for SHP2 inhibitors. We compared the sensitivity to SHP099 across 948 human tumor cell lines and 27 subsets of cancer. Interestingly, when subsets were analyzed as an entire group, HNSCC and the pediatric nervous system cancer neuroblastoma were the most sensitive subsets of cancer (Fig. 1A; Supplementary Table S1) (28). Follow-up crystal violet viability assays over 7 days confirmed sensitivity in HNSCC cells lines SCC-9 and JHU-022 and resistance in the HNSCC cell line BICR22 (Fig. 1B). Although SHP099 inhibition has been demonstrated to be specific to SHP2 through both profiling and mutational studies (29), we confirmed sensitivity profiles with RMC-4550 (19) a second, structurally unique allosteric inhibitor of SHP2 (Fig. 1C; Supplementary Fig. S1A and S1B).

As sensitivity to SHP099 is overall linked to inhibition of the MEK/ERK pathway (17), we determined whether the HNSCCs that were the most sensitive to SHP099 were also sensitive to the FDA-approved MEK inhibitor, trametinib. Interestingly, we did not find overlapping sensitivities (Fig. 1D; Supplementary Fig. S2A), which suggested to us that SHP099 may inhibit additional oncogenic pathways in sensitive HNSCC cell lines outside the MEK/ERK pathway. Of note, we also did not find overlapping sensitivities between SHP099 and the PI3K inhibitor, alpelisib (Supplementary Fig. S2B).

**FIGURE 2:**
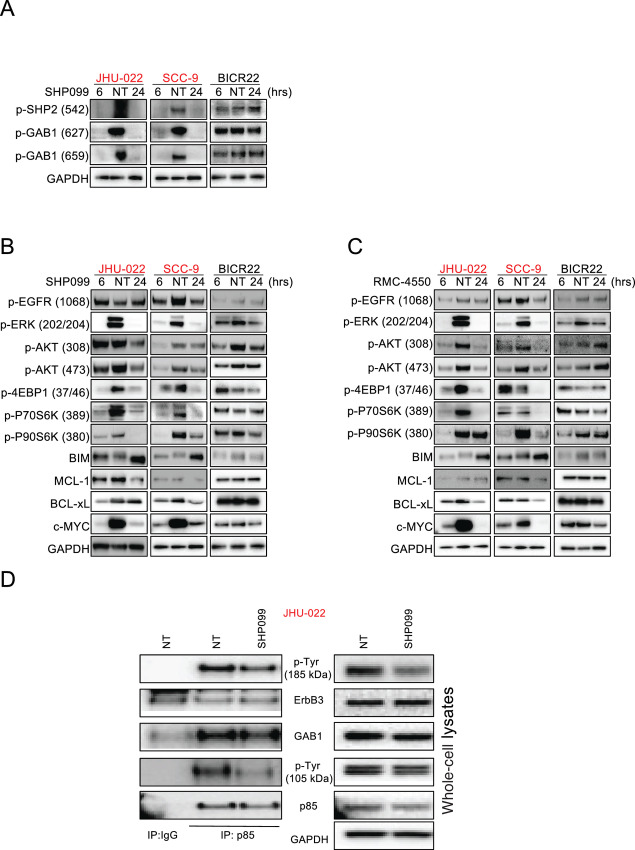
HNSCC cell lines are hypersensitive to SHP2 inhibition through dual PI3K and MEK inhibition. **A–C,** HNSCC cell lines were treated with 10 μmol/L SHP099 for 6 or 24 hours or 10 μmol/L RMC-4550 for 6 or 24 hours, as indicated, and extracted proteins were immunoblotted with the indicated antibodies. Please note, as identical lysates from the SCC-9 and BICR22 cells were used to probe the indicated antibodies in Fig. 2A and B, the same GAPDH blot served as the loading control and is shown twice. **D,** JHU-022 cells were treated with or without 10 μmol/L SHP099 for 6 hours and extracted proteins were subjected to (left) immunoprecipitation an anti-IgG antibody or an anti-p85 antibody and extracted proteins were immunoblotted with an anti-p-Tyr antibody, or (right) whole-cell lysates (5% input) were probed with the indicated antibodies in parallel. The blots were then stripped and reprobed with antibodies against the indicated proteins.

SHP2 can mediate both RAS/MEK/ERK activation and PI3K activation through Grb2-associated binder (GAB) proteins (30); how precisely SHP2 disruption leads to loss of RAS/MEK/ERK signaling remains unclear, but likely involves disruption of the multiprotein complex of GAB1/GRB2/SOS (19). GAB1 was initially identified as a docking protein phosphorylated in response to EGFR stimulation (31) and GAB1 has both an N-terminal plekstrin homology (PH) domain that is primarily responsible for the ability of EGFR to activate PI3K signaling, and two key pTYR sites (Y627 and Y659) that mediate SHP2-GAB1–driven activation of RAS/MEK/ERK (19, 21, 31, 32). In sensitive HNSCCs, but not insensitive HNSCCs, SHP2 inhibition led to near complete loss of GAB1 phosphorylation at the RAS activation sites Y627 and Y659, (Fig. 2A), consistent with loss of MEK/ERK signaling (Fig. 2B and C). In sensitive HNSCCs, we also noted SHP2 inhibitor treatment caused inhibition of PI3K, as evidenced by loss of pAKT (Fig. 2B and C), which has not been seen in previous studies in other contexts (18, 19, 33). Similarly, and consistent with the short-term experiments, we are able to be see SHP2 inhibitor suppressing both MEK/ERK and the PI3K/AKT pathway in longer experiments (Supplementary Fig. S3). We therefore sought the underlying mechanism. We immunoprecipitated (IP) p85 (regulatory subunit of PI3K), and probed p85 IP complexes with a pTYR antibody. In the corresponding whole-cell lysates, we found loss of pTYR caused by SHP099 in two proteins: one resolved at approximately 100 (consistent with the MW of GAB1), the second resolved at approximately 185 kDa, consistent with the ErbB family member and PI3K-activating protein, ErbB3 (ref. 34; Fig. 2D). We found identical loss of pTYR at 185 and 105 kDA, but more pronounced at 105 kDA in the IP (Fig. 2D, left; Supplementary Fig. S4A). Indeed, GAB1 and ErbB3 were detected at these molecular weights in the p85 IPs (Fig. 2D, left; Supplementary Fig. S4A). We confirmed pErbB3 reduction with an Ab raised against a specific Y residue (Y1328; Supplementary Fig. S4B). Other ErbB members at 185 kDA were also probed which are affected by SHP099 (Supplementary Fig. S5). But interestingly, we found that SHP099 affected the p85–GAB1 interaction but not the p85–ErbB3 interaction (Fig. 2D, left). These data suggest that SHP099-mediated loss of pTYR GAB1 is responsible for the inhibition of PI3K activity and not ERbB3.

**FIGURE 3:**
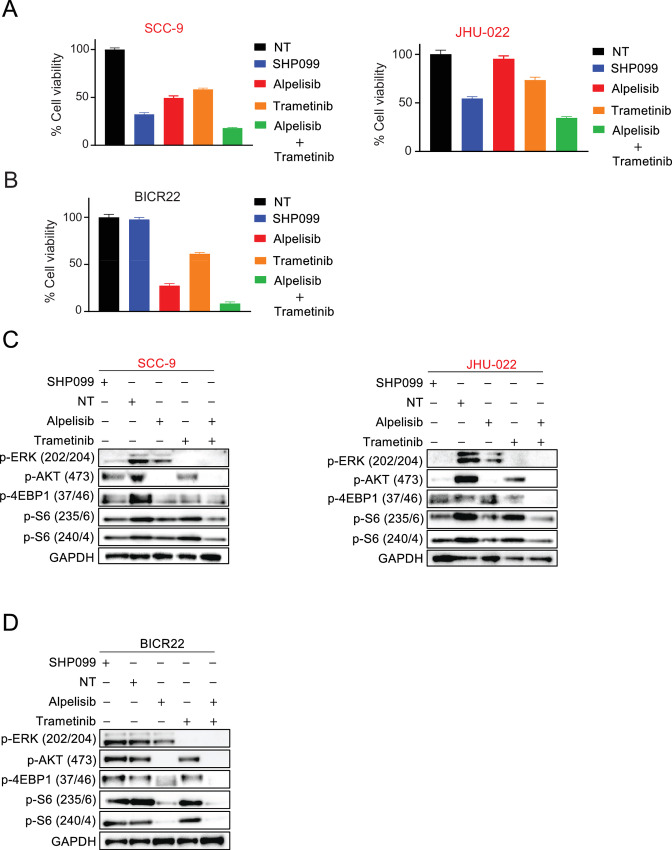
Comparison of SHP2 inhibitor SHP099, alpelisib and trametinib in SHP2- sensitive and -resistant HNSCC cell lines. **A** and **B**, SHP2 inhibitor–sensitive HNSCC cell lines SCC-9 and JHU-022 or SHP2 inhibitor-insensitive cells (BICR22) were treated with a fixed concentration of SHP099 (10 μmol/L), alpelisib (3 μmol/L), trametinib (10 nmol/L), or the combination for 3 days, and cell viability was determined by CellTiter-Glo. Extracted proteins from SCC-9 and JHU-022 cells (**C**) or BICR22 cells (**D**) treated with 10 μmol/L SHP099, 3 μmol/L alpelisib, 10 nmol/L trametinib or the combination for 6 hours were immunoblotted with the indicated antibodies.

**FIGURE 4:**
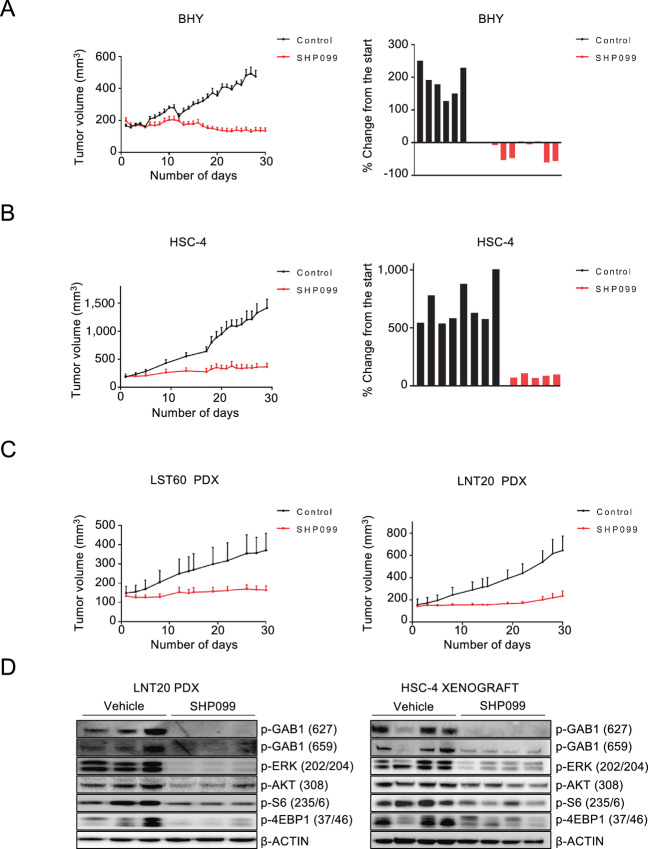
SHP099 is effective in mouse models of HNSCC. **A** and **B**, Left, Growth curves of the BHY and HSC-4 xenograft models. After tumor establishment (100–200 mm^3^), mice were treated with either vehicle (black lines) or administered 75 mg/kg of SHP099 (red lines) by oral gavage (6 days per week for 30 days). Right = waterfall plot of each tumor representing growth on days 28 and 29 for BHY tumors and HSC-4 tumors, respectively. **C,** Growth curves of the LST60 and LNT20 PDX models. Mice were treated with either vehicle (black) or administered 75 mg/kg of SHP099 (red). All error bars represent SEM. **D,** Left, Immunoblot of extracted proteins from the LNT20 PDX model treated with vehicle or SHP099 with the indicated antibodies. Right, Immunoblots from extracted proteins from HSC-4 xenografts treated with vehicle or SHP099.

**FIGURE 5:**
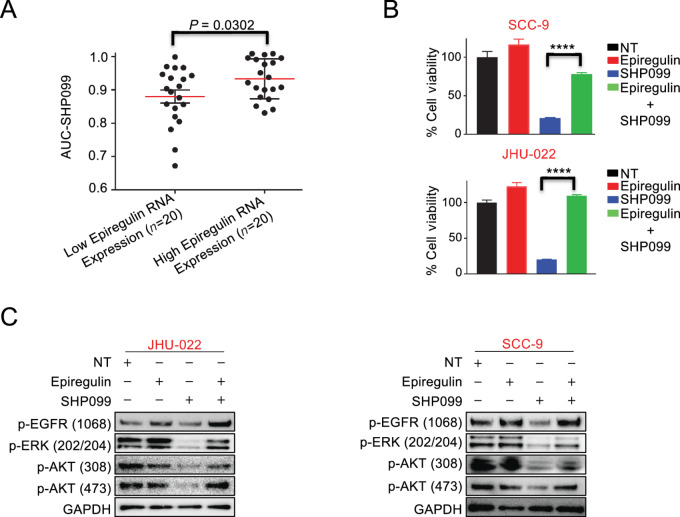
Role of EREG in maintaining mTOR activity upon SHP2 inhibitor SHP099 treatment. **A,** HNSCC cell lines cataloged in the GDSC database were clustered into low- and high-EREG RNA expression groups, and the SHP099 AUC data for these cell lines were plotted. The red bars indicate the means of each group. Statistical significance was determined by two-tailed Student *t* test. **B,** The viability of SCC-9 cells and JHU-022 cells treated for 7 days with or without 10 μmol/L SHP099 in the presence or absence of 50 ng/mL EREG. Statistical significance was determined using two-tailed Student *t* test. ****, *P* < 0.0001. **C,** Extracted proteins from JHU-022 cells treated for 24 hours and SCC-9 cells treated for 6 hours with 10 μmol/L SHP099 in the presence or absence of 50 ng/mL EREG were immunoblotted and probed with the indicated antibodies.

The PI3K and MEK/ERK pathways often converge on mTORC1 (25, 35). In SHP2 inhibitor–sensitive HNSCCs, we found mTORC1 was inhibited, as evidenced by loss of p70S6K and 4E-BP1 phosphorylation (ref. 35; Fig. 2B and C). In addition, the proapoptotic BIM, which is suppressed by the MEK/ERK/p90RSK pathway, was upregulated following SHP2 inhibition exclusively in the sensitive HNSCCs (Fig. 2B and C). Finally, we found, MCL-1, c-MYC, and BCL-xL expression, which are controlled tightly by the mTORC1 pathway (36, 37), were downregulated only in the sensitive HNSCCs (Fig. 2B and C). Therefore, in a subset of HNSCC, SHP099 blocks both MEK/ERK signaling and PI3K signaling, converging on mTORC1, likely explaining the potent anticancer activity of SHP2 inhibition.

### The Downregulation of Both the MEK and PI3K Pathway in HNSCC is Toxic

We and others (35, 38, 39) have found PI3K and MEK coinhibition has potent anticancer efficacy across multiple solid tumor paradigms. To confirm the therapeutic benefit of blocking both these pathways across HNSCC cell lines, we treated SHP2 inhibitor–sensitive JHU-022 and SCC-9 cells and SHP2 inhibitor–resistant BICR22 cells with the PI3K alpha inhibitor, alpelisib (40), and the MEK inhibitor, trametinib (41). As expected, the combination of alpelisib and trametinib was very potent against all three HNSCC cell lines (Fig. 3A and B) with alpelisib blocking and some TORC1 signaling in all three lines, trametinib blocking MEK signaling in all three lines, and the combination of the two robustly blocking mTORC1 signaling (Fig. 3C and D) in all three lines, consistent with our past work (38, 42) and others (15, 43). Also as expected, SHP2 inhibition blocked PI3K, MEK, and mTORC1 signaling (pS6) in the sensitive JHU-022 and SCC-9 cells, but not in the insensitive BICR22 cells. This led to similar efficacy between SHP099 and the alpelisib/trametinib combination in the SHP2 inhibitor–sensitive cells (Fig. 3A), and contrasting efficacy between SHP099 and the alpelisib/trametinib combination in the SHP2 inhibitor–resistance cells (Fig. 3B). These results are consistent with the anti-HNSCC activity of SHP099 occurring through coinhibition of MAPK and PI3K pathways, and these two pathways being the key targets of SHP2 inhibition in sensitive HNSCCs.

### HNSCC Cell Line and PDXs are Sensitive to SHP2 Inhibition

We next evaluated the efficacy of SHP099 *in vivo.* The BHY and HSC-4 xenograft models that presented with good sensitivity *in vitro* were injected to form tumors in immunocompromised mice. In addition, we recently established a collection of PDX models of HNSCC, including HPV+ models (27). We evaluated two of these models: a HPV− (LST60) and HPV+ model (LNT20). In keeping with the *in vitro* results, treatment with 75 mg/kg/qd SHP099 single agent led to near total tumor control across all models (Fig. 4A–C). Treatment was well tolerated on the basis of stable weight maintenance, normal behavior, and overall good health of the mice throughout the study (Supplementary Fig. S6, mouse weights). In the xenografts, the difference in tumor volume was visible as early as 10 days after the beginning of treatment with a consistent increase in difference between the two treatment arms throughout the study period (Fig. 4A and B, left). The waterfall diagrams show the differences in tumor volume after approximately 30 days of the study compared starting treatment volume demonstrating tumor regressions in most of the BHY-implanted mice and strong tumor control in the HSC-4 model (Fig. 4A and B, right). Similarly, the PDX models established from patients with HNSCC also revealed significant decreases in tumor burden upon treatment with SHP099 by the end of 30 days (Fig. 4C). In the tumor lysates, on-target activity, echoing the *in vitro* data of dual MEK and PI3K inhibition converging on mTORC1, was confirmed (Fig. 4D). These data together validate the sensitivity demonstrated in the unbiased HTS screen of a large number of HNSCC models that have sensitivity to SHP2 inhibition.

**FIGURE 6:**
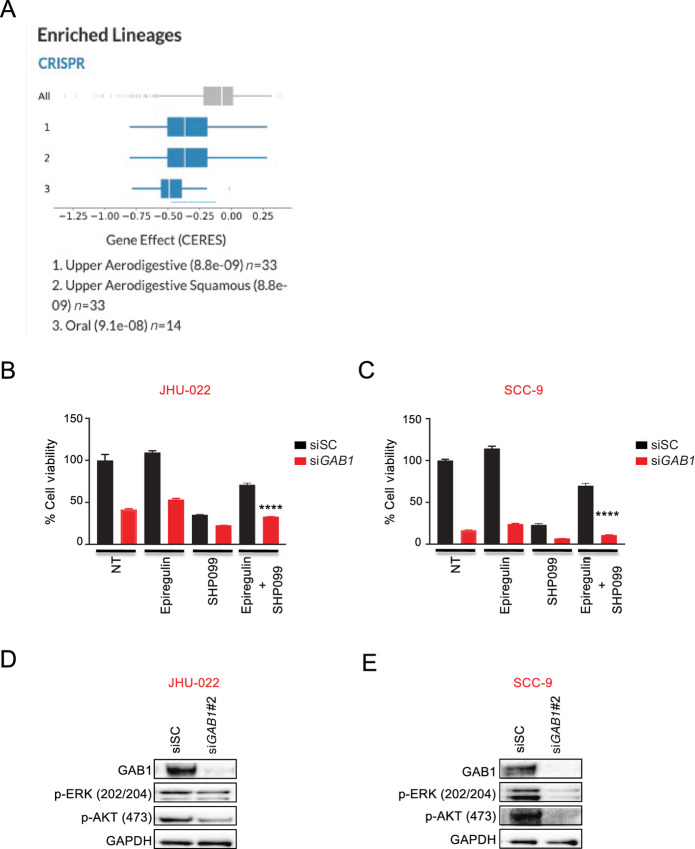
HNSCC are dependent on the GAB1 gene and GAB1 knockdown results in inhibition of cell proliferation and intracellular oncogenic signaling in HNSCC. **A,** HNSCC cell lines are dependent on the GAB1 gene as determined by the CERES dependency score following CRISPR-Cas9–based viability screens (DepMap consortium). A low score indicates high gene dependency. Please note: the top three subsets are HNSCC. **B** and **C,** JHU-022 and SCC-9 cells were transiently transfected with either scrambled (SC) or GAB1 siRNA for 24 hours, reseeded, and the next day the cells were treated with or without 10 μmol/L SHP099 in the presence or absence of 50 ng/mL EREG for 3 days, and cell viability was determined by CellTiter-Glo. Statistical significance was determined using two-tailed Student *t* test. ****, *P* < 0.0001. **D** and **E**, JHU-022 and SCC-9 cells were transiently transfected with either GAB1 or scrambled (SC) siRNA for 48 hours and extracted proteins were subjected to Western blot analyses with the indicated antibodies.

### Expression of the EGF Ligand EREG Correlates with Response to SHP099 in HNSCC

We next sought to understand which HNSCCs made up the sensitive group which could potentially enable biomarker-directed clinical trials in HNSCC and to also better understand the mechanism of SHP2 inhibitor efficacy in HNSCC. As HNSCCs are void of activating mutations in RTKs (44), ErbB ligands are important mediators of RTK signaling in HNSCC, particularly those that activate the large number of EGF receptors in these cancers (45). We therefore investigated the most prominent ErbB ligands, including EGF, EREG, neuregulin-1, and amphiregulin. While expression of most of these ligands had no predictive power (Supplementary Fig. S7), low-EREG HNSCCs were significantly more sensitive to SHP099, both by median expression and by the lowest AUC (Fig. 5A). On the basis of these data, we added exogenous EREG to sensitive HNSCC cell lines, to determine whether this was sufficient to confer resistance. Strikingly, exogenous EREG was sufficient to substantially mitigate SHP099 efficacy across both sensitive models (JHU-22 and SCC9) tested (Fig. 5B). Consistently, Western blot analyses demonstrated in these cell lines that the presence of EREG led to the maintenance of MEK and PI3K signaling and activation of EGFR following SHP099 therapy (Fig. 5C). This contrasted with the addition of other ErbB family ligands like neuregulin-1, which consistent with the lack of predictive power, did not alter the ability of SHP099 to block PI3K or MEK signaling or exert anti-HNSCC activity (Supplementary Fig. S8A and S8B). Altogether, these data demonstrate that SHP099 blocks MEK/ERK and PI3K signaling when EREG levels are low, conferring sensitivity to the drug, and sustained MEK/ERK and PI3K signaling when EREG levels are high confers resistance to SHP2 inhibition.

**FIGURE 7:**
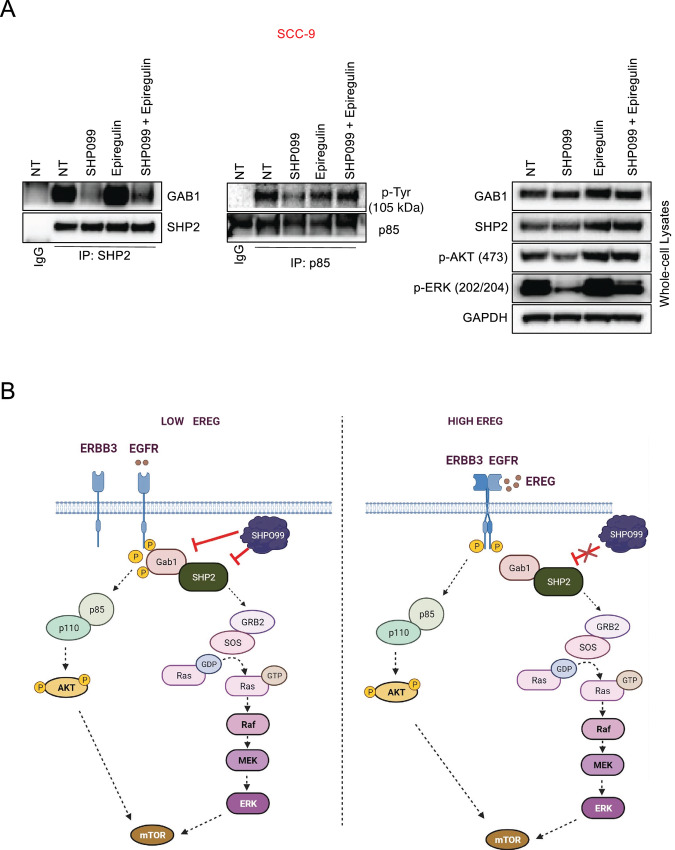
Model of SHP099 inhibitor efficacy and the role of EREG in HNSCC. **A,** SCC-9 cells were treated with or without 10 μmol/L SHP099 in the presence or absence of 50 ng/mL EREG for 6 hours. Cell lysates were subjected to immunoprecipitation with (left) an anti-IgG antibody or an SHP2 antibody and an anti-IgG antibody or anti-p85 antibody (center) followed by Western blot analysis with an GAB1 or an anti-p-Tyr antibody or (right) whole-cell lysates (5% input) were probed with the indicated antibodies in parallel. The blots were then stripped and reprobed with antibodies against the indicated proteins. **B,** In low-EREG HNSCC, SHP2 inhibition is effective by disrupting SHP2–GAB1 complexes and PI3K and MEK signaling, leading to downregulation of mTOR and anti-HNSCC effects. In high-EREG HNSCC, EREG mitigates SHP2 inhibitor by maintaining PI3K signaling. This is likely largely independent of SHP2, and therefore SHP2 inhibition is not sufficient to block PI3K signaling.

### GAB1 is a Critical Protein in HNSCC Survival, and Regulates EREG-mediated Rescue of SHP2 Inhibitor Toxicity

Because of the role of GAB1 in SHP2 inhibitor efficacy (Fig. 2), we examined further a possible role in SHP2 inhibitor toxicity and subsequent EREG rescue. Using the DepMap database (46), we found among approximately 1,300 cancer cell lines, HNSCC cell lines were the most sensitive to GAB1 genetic editing (Fig. 6A). Analyses of gene dependencies found GAB1 and EGFR were most closely related, highlighting the importance of GAB1 for EGFR-driven cancers (Supplementary Fig. S9A), like HNSCC.

We next genetically silenced GAB1 to directly probe its role in SHP2 inhibitor efficacy. Of note, over 3 days, GAB1 knockdown markedly blocked the growth of both the JHU-22 and SCC-9 cells (Fig. 6B and C; Supplementary Fig. S9B and S9C) and was particularly toxic in the SCC-9 cells. To assess whether GAB1 plays a causative role in mediating resistance to SHP2 inhibitor–resistant BICR22 cells, we silenced GAB1. Knockdown of GAB1 did not impart SHP099 sensitivity (Supplementary Fig. S10A and S10B). Biochemical analyses revealed that knockdown of GAB1, similar to pharmaceutical inhibition of SHP2 (Figs. 2B, 2C, 3C, 3D, and 4D), was sufficient to block both PI3K and MEK signaling in both cell lines, which consistent with the efficacy data, was more marked in the SCC-9 cells compared with JHU-022 cells (Fig. 6D and E). While EREG was again sufficient to exert a small but measurable proliferative advantage over the no drug treatment controls in both the si scramble (SC) and siGAB1-transfected cells [Figs. 6B and C (3 days), compare with Fig. 5B (7 days)], and SHP099 efficacy was again largely mitigated by the presence of EREG in the siSC-treated cells (Fig. 6B and C, compare with Fig. 5B), the siGAB1-treated cells were refractory to EREG rescue (Fig. 6B and C).

SHP2 complexes with GAB1 to affect PI3K and RAS/MEK signaling (20–22, 46). Our demonstration that GAB1 is a critical mediator of both PI3K and MEK signaling and SHP099 efficacy in HNSCC alerted us to examine more closely the interaction of GAB1 and SHP2 and how EREG may alter this complex. Assessment of SHP2–GAB1 complexes revealed while SHP099 expectedly disrupted this complex, the presence of exogenous EREG increased complexed SHP2 and GAB1, and, importantly, led to the maintenance SHP2–GAB1 complexes, in parallel with maintenance of pERK and pAKT signaling in the whole-cell lysates (Fig. 7A). Overall, these data demonstrate that GAB1 regulates the EREG-mediated rescue of both the PI3K and MEK pathways in HNSCC and is a critical survival protein in HNSCC (Fig. 7B).

## Discussion

In this study, utilizing our HTS platform, we identified sensitivity to HNSCCs to SHP2 inhibition. SHP099 has been demonstrated to be effective in a number of preclinical solid tumor models, particularly in combinations that result in enhanced and/or prolonged MEK/ERK inhibition (17, 47). The unexpected lack of overlap of SHP2 inhibitor sensitivity and MEK inhibitor sensitivity in HNSCC led us to investigate whether other pathways in addition to the MEK pathway were being blocked by SHP099. Indeed, we found robust inhibition of the PI3K pathway in HNSCCs that were sensitive to SHP099. Neel and colleagues demonstrated that in different contexts, based on signaling flux through RTKs, SHP2 can either block or potentiate PI3K signaling (48). Our data demonstrate that SHP2 inhibition disrupts GAB1-p85 binding to downregulate PI3K signaling in HNSCC. GAB1 can be an important mediator of EGFR-PI3K activation (32).

Preclinically, we (38, 49, 50) and others (11, 35, 39, 51) have demonstrated the dual combination of MEK and PI3K signaling is highly efficacious across a number of different solid tumors; unfortunately, clinically, this combination has been too toxic to achieve doses which the drugs can achieve target inhibition. In successful RTK targeted therapy paradigms, like EGFR inhibitors in *EGFR*-mutant NSCLC, sensitivity is conferred because EGFR inhibitors lead to simultaneous downregulation of PI3K and MEK pathways; restoration of either pathway is sufficient to confer resistance (13, 15).

EGF family ligands are important activators of EGFR signaling in diverse cancers (45). Among the EGF ligand family is EREG, which is found at higher levels in HNSCCs than normal gingivae, increases in expression as HNSCCs advance, and portend poor outcomes (52). In HNSCC, EGF family ligand expression, including EREG, associates with better responses to the EGFR inhibitor cetuximab, reflecting the increased dependence on EGFR signaling in these cancers (53). Indeed, we found EREG increased EGFR activation in our study. In contrast to cetuximab, we show SHP2 inhibitor is effective when EREG levels are low, correlating with the ability of SHP099 to sufficiently block both MEK and PI3K pathways. These data were also replicated with the allosteric SHP2 inhibitor, RMC-4550. Consistent with these data, exogenous expression of EREG was sufficient to prevent SHP099-mediated downregulation of PI3K and MEK pathways, and as such, to mitigate SHP099 efficacy. These data suggest a flux-dependent SHP2 inhibitor effect on these pathways and a molecular biomarker for SHP2 inhibitor clinical trials. Of importance, EREG has been demonstrated to be assessible as a biomarker in colorectal cancer, suggesting clinical implementation is achievable (54).

We have found GAB1 is critical for HNSCC survival and plays an important role in mediating the response of SHP2 inhibition. GAB1 is a PH-containing scaffolding protein for diverse RTKs and binds both SH2 and SH3 domains (55). These data are consistent with the correlation we found between EGFR and GAB1 in the Depmap CRISPR/Cas9 screening data and fits the picture that targeting GAB1 complexes—for instance, by targeting SHP2—may be a more potent therapy for HNSCC than EGFR inhibition. Our model therefore is that when EREG is low, SHP2 can mediate both RAS/MEK/ERK activation and PI3K activation through GAB proteins. The relative lack of flux through the EGFR receptor allows for robust inhibition of PI3K and MEK pathways following SHP2 inhibition. When EREG is high, there is reactivation of GAB1–P85 complexes, which contributes to reactivation of downstream pathways (Fig. 7B). In addition, EREG also leads to the activation of ErbB3 (56), and ErbB3, like GAB1, can also activate PI3K (34), which could further contribute the resistance phenotype we detect with EREG. EREG ligand and other EGFR family ligands and their role in SHP2 inhibitor response. Additionally, both combined EGFR and SHP2 or combined ErbB3 and SHP2 inhibition may offer superior reduction of PI3K and ERK pathways. We are investigating these combinations currently in our laboratories.

While we did not observe overt toxicity in our studies, comprehensive toxicity of SHP2 inhibitors was not well characterized in this study. SHP2 inhibitors are being evaluated in clinical trials. Important toxicity data will be available soon from these trials, offering insight into a potential therapeutic window of this new class of drugs (28).

HNSCCs continue to be treated with radiation and high-dose platinum-based therapies, and these treatments are accompanied by a high rate of morbidity. Here, we demonstrate SHP2 inhibitors can downregulate both PI3K and MEK pathways in HNSCC cell lines and are effective in a large subset of HNSCCs. Consistently, SHP099 blocks the growth of HNSCC xenograft models as well as PDX models, including an HPV+ model. While there may be biological differences that underlie differential sensitivity in HPV+ HNSCCs versus HPV− HNSCCs, a much more thorough investigation including numerous models will be needed to determine whether this is so. Our findings in total suggest that inhibition of SHP2 could have clinical utility in HNSCC, sensitive HNSCCs can be identified by expression levels of EREG, and SHP2 inhibition leads to downregulation of both MEK and PI3K pathways in sensitive HNSCCs. Several SHP2 inhibitors are now in clinical evaluation. SHP2 inhibitors as monotherapy or a potentiator of current HNSCC therapies warrants further consideration.

## Supplementary Material

Figure S1 Cell viability of SHP099-sensitive HNSCC cell lines

Figure S2 HNSCC tumor cell lines are effective to SHP099 through dual PI3K and MEK inhibition

Figure S3 Three- and five-day western blot analysis of SHP099-sensitive and - resistant HNSCC cell lines

Figure S4 The mechanism of action of SHP099 inhibition

Figure S5 Western blot analysis of ErbB members

Figure S6 Weight profiles of xenograft models treated with SHP099

Figure S7 RTK ligand expression in HNSCC cell lines

Figure S8 Neuregulin-1 does not rescue PI3K and MEK signaling in HNSCC treated with SHP2 inhibitor

Figure S9 Biological importance of Gab1 in HNSCC cells

Figure S10 Effects of knockdown of Gab1 in SHP099-resistant HNSCC cell line

Table S1 Cell line information and SHP099 screen data

## References

[1] Mourad M , Jetmore T, Jategaonkar AA, Moubayed S, Moshier E, Urken ML. Epidemiological trends of head and neck cancer in the united states: a SEER population study. J Oral Maxillofac Surg 2017;75:2562–72. 28618252 10.1016/j.joms.2017.05.008 PMC6053274

[2] Rothenberg SM , Ellisen LW. The molecular pathogenesis of head and neck squamous cell carcinoma. J Clin Invest 2012;122:1951–7. 22833868 10.1172/JCI59889 PMC3589176

[3] Mehanna H , Beech T, Nicholson T, El-Hariry I, McConkey C, Paleri V, . Prevalence of human papillomavirus in oropharyngeal and nonoropharyngeal head and neck cancer–systematic review and meta-analysis of trends by time and region. Head Neck 2013;35:747–55. 22267298 10.1002/hed.22015

[4] Chung CH , Parker JS, Karaca G, Wu J, Funkhouser WK, Moore D, . Molecular classification of head and neck squamous cell carcinomas using patterns of gene expression. Cancer Cell 2004;5:489–500. 15144956 10.1016/s1535-6108(04)00112-6

[5] Harrington KJ , Ferris RL, Blumenschein G Jr, Colevas AD, Fayette J, Licitra L, . Nivolumab versus standard, single-agent therapy of investigator's choice in recurrent or metastatic squamous cell carcinoma of the head and neck (CheckMate 141): health-related quality-of-life results from a randomised, phase 3 trial. Lancet Oncol 2017;18:1104–15. 28651929 10.1016/S1470-2045(17)30421-7 PMC6461049

[6] Chow LQM , Haddad R, Gupta S, Mahipal A, Mehra R, Tahara M, . Antitumor activity of pembrolizumab in biomarker-unselected patients with recurrent and/or metastatic head and neck squamous cell carcinoma: results from the phase Ib KEYNOTE-012 expansion cohort. J Clin Oncol 2016;34:3838–45. 27646946 10.1200/JCO.2016.68.1478 PMC6804896

[7] Malone E , Siu LL. Precision medicine in head and neck cancer: myth or reality? Clin Med Insights Oncol 2018;12:1179554918779581. 29887732 10.1177/1179554918779581 PMC5989049

[8] Bonner JA , Harari PM, Giralt J, Cohen RB, Jones CU, Sur RK, . Radiotherapy plus cetuximab for locoregionally advanced head and neck cancer: 5-year survival data from a phase 3 randomised trial, and relation between cetuximab-induced rash and survival. Lancet Oncol 2010;11:21–8. 19897418 10.1016/S1470-2045(09)70311-0

[9] Vermorken JB , Mesia R, Rivera F, Remenar E, Kawecki A, Rottey S, . Platinum-based chemotherapy plus cetuximab in head and neck cancer. N Engl J Med 2008;359:1116–27. 18784101 10.1056/NEJMoa0802656

[10] Reeves TD , Hill EG, Armeson KE, Gillespie MB. Cetuximab therapy for head and neck squamous cell carcinoma: a systematic review of the data. Otolaryngol Head Neck Surg 2011;144:676–84. 21493327 10.1177/0194599811399559

[11] Engelman JA , Chen L, Tan X, Crosby K, Guimaraes AR, Upadhyay R, . Effective use of PI3K and MEK inhibitors to treat mutant Kras G12D and PIK3CA H1047R murine lung cancers. Nat Med 2008;14:1351–6. 19029981 10.1038/nm.1890 PMC2683415

[12] Alagesan B , Contino G, Guimaraes AR, Corcoran RB, Deshpande V, Wojtkiewicz GR, . Combined MEK and PI3K inhibition in a mouse model of pancreatic cancer. Clin Cancer Res 2015;21:396–404. 25348516 10.1158/1078-0432.CCR-14-1591 PMC4447091

[13] Niederst MJ , Engelman JA. Bypass mechanisms of resistance to receptor tyrosine kinase inhibition in lung cancer. Sci Signal 2013;6:re6. 24065147 10.1126/scisignal.2004652 PMC3876281

[14] Chapman PB , Hauschild A, Robert C, Haanen JB, Ascierto P, Larkin J, . Improved survival with vemurafenib in melanoma with BRAF V600E mutation. N Engl J Med 2011;364:2507–16. 21639808 10.1056/NEJMoa1103782 PMC3549296

[15] Serra V , Scaltriti M, Prudkin L, Eichhorn PJ, Ibrahim YH, Chandarlapaty S, . PI3K inhibition results in enhanced HER signaling and acquired ERK dependency in HER2-overexpressing breast cancer. Oncogene 2011;30:2547–57. 21278786 10.1038/onc.2010.626 PMC3107390

[16] Mendoza MC , Er EE, Blenis J. The Ras-ERK and PI3K-mTOR pathways: cross-talk and compensation. Trends Biochem Sci 2011;36:320–8. 21531565 10.1016/j.tibs.2011.03.006 PMC3112285

[17] Torres-Ayuso P , Brognard J. Shipping out MEK inhibitor resistance with SHP2 inhibitors. Cancer Discov 2018;8:1210–2. 30279193 10.1158/2159-8290.CD-18-0915

[18] Dardaei L , Wang HQ, Singh M, Fordjour P, Shaw KX, Yoda S, . SHP2 inhibition restores sensitivity in ALK-rearranged non-small-cell lung cancer resistant to ALK inhibitors. Nat Med 2018;24:512–7. 29505033 10.1038/nm.4497 PMC6343825

[19] Nichols RJ , Haderk F, Stahlhut C, Schulze CJ, Hemmati G, Wildes D, . RAS nucleotide cycling underlies the SHP2 phosphatase dependence of mutant BRAF-, NF1- and RAS-driven cancers. Nat Cell Biol 2018;20:1064–73. 30104724 10.1038/s41556-018-0169-1 PMC6115280

[20] Zhang RY , Yu ZH, Chen L, Walls CD, Zhang S, Wu L, . Mechanistic insights explain the transforming potential of the T507K substitution in the protein-tyrosine phosphatase SHP2. J Biol Chem 2020;295:6187–201. 32188694 10.1074/jbc.RA119.010274 PMC7196634

[21] Montagner A , Yart A, Dance M, Perret B, Salles JP, Raynal P. A novel role for Gab1 and SHP2 in epidermal growth factor-induced Ras activation. J Biol Chem 2005;280:5350–60. 15574420 10.1074/jbc.M410012200

[22] Fragale A , Tartaglia M, Wu J, Gelb BD. Noonan syndrome-associated SHP2/PTPN11 mutants cause EGF-dependent prolonged GAB1 binding and sustained ERK2/MAPK1 activation. Hum Mutat 2004;23:267–77. 14974085 10.1002/humu.20005

[23] Iorio F , Knijnenburg TA, Vis DJ, Bignell GR, Menden MP, Schubert M, . A landscape of pharmacogenomic interactions in cancer. Cell 2016;166:740–54. 27397505 10.1016/j.cell.2016.06.017 PMC4967469

[24] Chen YN , LaMarche MJ, Chan HM, Fekkes P, Garcia-Fortanet J, Acker MG, . Allosteric inhibition of SHP2 phosphatase inhibits cancers driven by receptor tyrosine kinases. Nature 2016;535:148–52. 27362227 10.1038/nature18621

[25] Haagensen EJ , Kyle S, Beale GS, Maxwell RJ, Newell DR. The synergistic interaction of MEK and PI3K inhibitors is modulated by mTOR inhibition. Br J Cancer 2012;106:1386–94. 22415236 10.1038/bjc.2012.70 PMC3326670

[26] Garnett MJ , Edelman EJ, Heidorn SJ, Greenman CD, Dastur A, Lau KW, . Systematic identification of genomic markers of drug sensitivity in cancer cells. Nature 2012;483:570–5. 22460902 10.1038/nature11005 PMC3349233

[27] Facompre ND , Sahu V, Montone KT, Harmeyer KM, Nakagawa H, Rustgi AK, . Barriers to generating PDX models of HPV-related head and neck cancer. Laryngoscope 2017;127:2777–83. 28561270 10.1002/lary.26679 PMC5687999

[28] Cai J , Jacob S, Kurupi R, Dalton KM, Coon C, Greninger P, . High-risk neuroblastoma with NF1 loss of function is targetable using SHP2 inhibition. Cell Rep 2022;40:111095. 35905710 10.1016/j.celrep.2022.111095 PMC10353975

[29] Ran H , Tsutsumi R, Araki T, Neel BG. Sticking it to cancer with molecular glue for SHP2. Cancer Cell 2016;30:194–6. 27505669 10.1016/j.ccell.2016.07.010 PMC5558882

[30] Sampaio C , Dance M, Montagner A, Edouard T, Malet N, Perret B, . Signal strength dictates phosphoinositide 3-kinase contribution to Ras/extracellular signal-regulated kinase 1 and 2 activation via differential Gab1/Shp2 recruitment: consequences for resistance to epidermal growth factor receptor inhibition. Mol Cell Biol 2008;28:587–600. 18025104 10.1128/MCB.01318-07 PMC2223412

[31] Holgado-Madruga M , Emlet DR, Moscatello DK, Godwin AK, Wong AJ. A Grb2-associated docking protein in EGF- and insulin-receptor signalling. Nature 1996;379:560–4. 8596638 10.1038/379560a0

[32] Rodrigues GA , Falasca M, Zhang Z, Ong SH, Schlessinger J. A novel positive feedback loop mediated by the docking protein Gab1 and phosphatidylinositol 3-kinase in epidermal growth factor receptor signaling. Mol Cell Biol 2000;20:1448–59. 10648629 10.1128/mcb.20.4.1448-1459.2000 PMC85307

[33] Pádua RAP , Sun Y, Marko I, Pitsawong W, Stiller JB, Otten R, . Mechanism of activating mutations and allosteric drug inhibition of the phosphatase SHP2. Nat Commun 2018;9:4507. 30375376 10.1038/s41467-018-06814-w PMC6207724

[34] Guix M , Faber AC, Wang SE, Olivares MG, Song Y, Qu S, . Acquired resistance to EGFR tyrosine kinase inhibitors in cancer cells is mediated by loss of IGF-binding proteins. J Clin Invest 2008;118:2609–19. 18568074 10.1172/JCI34588 PMC2430495

[35] She QB , Halilovic E, Ye Q, Zhen W, Shirasawa S, Sasazuki T, . 4E-BP1 is a key effector of the oncogenic activation of the AKT and ERK signaling pathways that integrates their function in tumors. Cancer Cell 2010;18:39–51. 20609351 10.1016/j.ccr.2010.05.023 PMC3286650

[36] Faber AC , Coffee EM, Costa C, Dastur A, Ebi H, Hata AN, . mTOR inhibition specifically sensitizes colorectal cancers with KRAS or BRAF mutations to BCL-2/BCL-XL inhibition by suppressing MCL-1. Cancer Discov 2014;4:42–52. 24163374 10.1158/2159-8290.CD-13-0315 PMC3973435

[37] Ma XM , Blenis J. Molecular mechanisms of mTOR-mediated translational control. Nat Rev Mol Cell Biol 2009;10:307–18. 19339977 10.1038/nrm2672

[38] Faber AC , Li D, Song Y, Liang MC, Yeap BY, Bronson RT, . Differential induction of apoptosis in HER2 and EGFR addicted cancers following PI3K inhibition. Proc Natl Acad Sci U S A 2009;106:19503–8. 19850869 10.1073/pnas.0905056106 PMC2765921

[39] Will M , Qin AC, Toy W, Yao Z, Rodrik-Outmezguine V, Schneider C, . Rapid induction of apoptosis by PI3K inhibitors is dependent upon their transient inhibition of RAS-ERK signaling. Cancer Discov 2014;4:334–47. 24436048 10.1158/2159-8290.CD-13-0611 PMC4049524

[40] Stirrups R . Alpelisib plus fulvestrant for PIK3CA-mutated breast cancer. Lancet Oncol 2019;20:e347. 31130321 10.1016/S1470-2045(19)30372-9

[41] Long GV , Stroyakovskiy D, Gogas H, Levchenko E, de Braud F, Larkin J, . Dabrafenib and trametinib versus dabrafenib and placebo for Val600 BRAF-mutant melanoma: a multicentre, double-blind, phase 3 randomised controlled trial. Lancet 2015;386:444–51. 26037941 10.1016/S0140-6736(15)60898-4

[42] Faber AC , Corcoran RB, Ebi H, Sequist LV, Waltman BA, Chung E, . BIM expression in treatment-naive cancers predicts responsiveness to kinase inhibitors. Cancer Discov 2011;1:352–65. 22145099 10.1158/2159-8290.CD-11-0106 PMC3229203

[43] Jokinen E , Koivunen JP. MEK and PI3K inhibition in solid tumors: rationale and evidence to date. Ther Adv Med Oncol 2015;7:170–80. 26673580 10.1177/1758834015571111 PMC4406912

[44] Hammerman PS , Hayes DN, Grandis JR. Therapeutic insights from genomic studies of head and neck squamous cell carcinomas. Cancer Discov 2015;5:239–44. 25643909 10.1158/2159-8290.CD-14-1205 PMC4355279

[45] Schlessinger J . Ligand-induced, receptor-mediated dimerization and activation of EGF receptor. Cell 2002;110:669–72. 12297041 10.1016/s0092-8674(02)00966-2

[46] McFarland JM , Ho ZV, Kugener G, Dempster JM, Montgomery PG, Bryan JG, . Improved estimation of cancer dependencies from large-scale RNAi screens using model-based normalization and data integration. Nat Commun 2018;9:4610. 30389920 10.1038/s41467-018-06916-5 PMC6214982

[47] Ahmed TA , Adamopoulos C, Karoulia Z, Wu X, Sachidanandam R, Aaronson SA, . SHP2 drives adaptive resistance to ERK signaling inhibition in molecularly defined subsets of ERK-dependent tumors. Cell Rep 2019;26:65–78. 30605687 10.1016/j.celrep.2018.12.013 PMC6396678

[48] Zhang SQ , Tsiaras WG, Araki T, Wen G, Minichiello L, Klein R, . Receptor-specific regulation of phosphatidylinositol 3'-kinase activation by the protein tyrosine phosphatase Shp2. Mol Cell Biol 2002;22:4062–72. 12024020 10.1128/MCB.22.12.4062-4072.2002 PMC133866

[49] Ebi H , Costa C, Faber AC, Nishtala M, Kotani H, Juric D, . PI3K regulates MEK/ERK signaling in breast cancer via the Rac-GEF, P-Rex1. Proc Nat Acad Sci U S A 2013;110:21124–9. 10.1073/pnas.1314124110 PMC3876254 24327733

[50] Roper J , Sinnamon MJ, Coffee EM, Belmont P, Keung L, Georgeon-Richard L, . Combination PI3K/MEK inhibition promotes tumor apoptosis and regression in PIK3CA wild-type, KRAS mutant colorectal cancer. Cancer Lett 2014;347:204–11. 24576621 10.1016/j.canlet.2014.02.018 PMC4118771

[51] Garcia-Garcia C , Rivas MA, Ibrahim YH, Calvo MT, Gris-Oliver A, Rodriguez O, . MEK plus PI3K/mTORC1/2 therapeutic efficacy is impacted by TP53 mutation in preclinical models of colorectal cancer. Clin Cancer Res 2015;21:5499–510. 26272063 10.1158/1078-0432.CCR-14-3091 PMC5087596

[52] Shigeishi H , Higashikawa K, Hiraoka M, Fujimoto S, Mitani Y, Ohta K, . Expression of epiregulin, a novel epidermal growth factor ligand associated with prognosis in human oral squamous cell carcinomas. Oncol Rep 2008;19:1557–64. 18497965

[53] Kogashiwa Y , Inoue H, Kuba K, Araki R, Yasuda M, Nakahira M, . Prognostic role of epiregulin/amphiregulin expression in recurrent/metastatic head and neck cancer treated with cetuximab. Head Neck 2018;40:2424–31. 30302873 10.1002/hed.25353

[54] Jacobs B , De Roock W, Piessevaux H, Van Oirbeek R, Biesmans B, De Schutter J, . Amphiregulin and epiregulin mRNA expression in primary tumors predicts outcome in metastatic colorectal cancer treated with cetuximab. J Clin Oncol 2009;27:5068–74. 19738126 10.1200/JCO.2008.21.3744

[55] Wang W , Xu S, Yin M, Jin ZG. Essential roles of Gab1 tyrosine phosphorylation in growth factor-mediated signaling and angiogenesis. Int J Cardiol 2015;181:180–4. 25528308 10.1016/j.ijcard.2014.10.148 PMC4385407

[56] Liu S , Wang Y, Han Y, Xia W, Zhang L, Xu S, . EREG-driven oncogenesis of head and neck squamous cell carcinoma exhibits higher sensitivity to erlotinib therapy. Theranostics 2020;10:10589–605. 32929368 10.7150/thno.47176 PMC7482801

