# Supplementary material for: Pharmacologic Inhibition of SHP2 Blocks Both PI3K and MEK Signaling in Low-epiregulin HNSCC via GAB1
Source: Cancer Res Commun. 2022 Sep 26;2(9):1061–74. doi: 10.1158/2767-9764.CRC-21-0137 (PMC9728803; doi:10.1158/2767-9764.CRC-21-0137)
Supplement: Figure S1 — Cell viability of SHP099-sensitive HNSCC cell lines [file crc-21-0137-s01.pptx]

## Slide 1
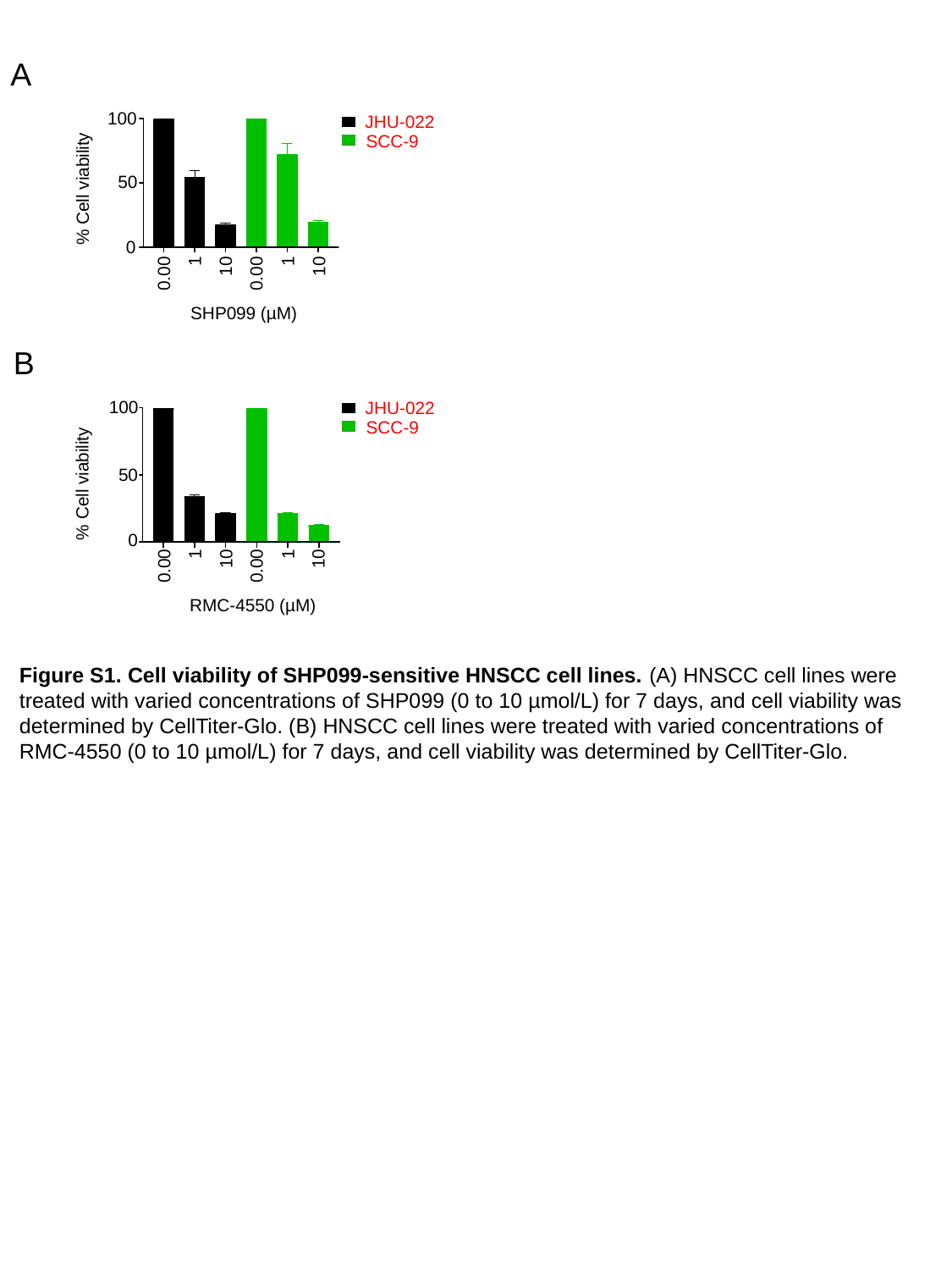

A
100
JHU-022
SCC-9
% Cell viability
50
0
1
10
1
10
0.00
0.00
SHP099 (µM)
B
100
JHU-022
SCC-9
% Cell viability
50
0
1
10
1
10
0.00
0.00
RMC-4550 (µM)
Figure S1. Cell viability of SHP099-sensitive HNSCC cell lines. (A) HNSCC cell lines were treated with varied concentrations of SHP099 (0 to 10 µmol/L) for 7 days, and cell viability was determined by CellTiter-Glo. (B) HNSCC cell lines were treated with varied concentrations of RMC-4550 (0 to 10 µmol/L) for 7 days, and cell viability was determined by CellTiter-Glo.
