# Supplementary material for: Pharmacologic Inhibition of SHP2 Blocks Both PI3K and MEK Signaling in Low-epiregulin HNSCC via GAB1
Source: Cancer Res Commun. 2022 Sep 26;2(9):1061–74. doi: 10.1158/2767-9764.CRC-21-0137 (PMC9728803; doi:10.1158/2767-9764.CRC-21-0137)
Supplement: Figure S2 — HNSCC tumor cell lines are effective to SHP099 through dual PI3K and MEK inhibition [file crc-21-0137-s02.pptx]

## Slide 1
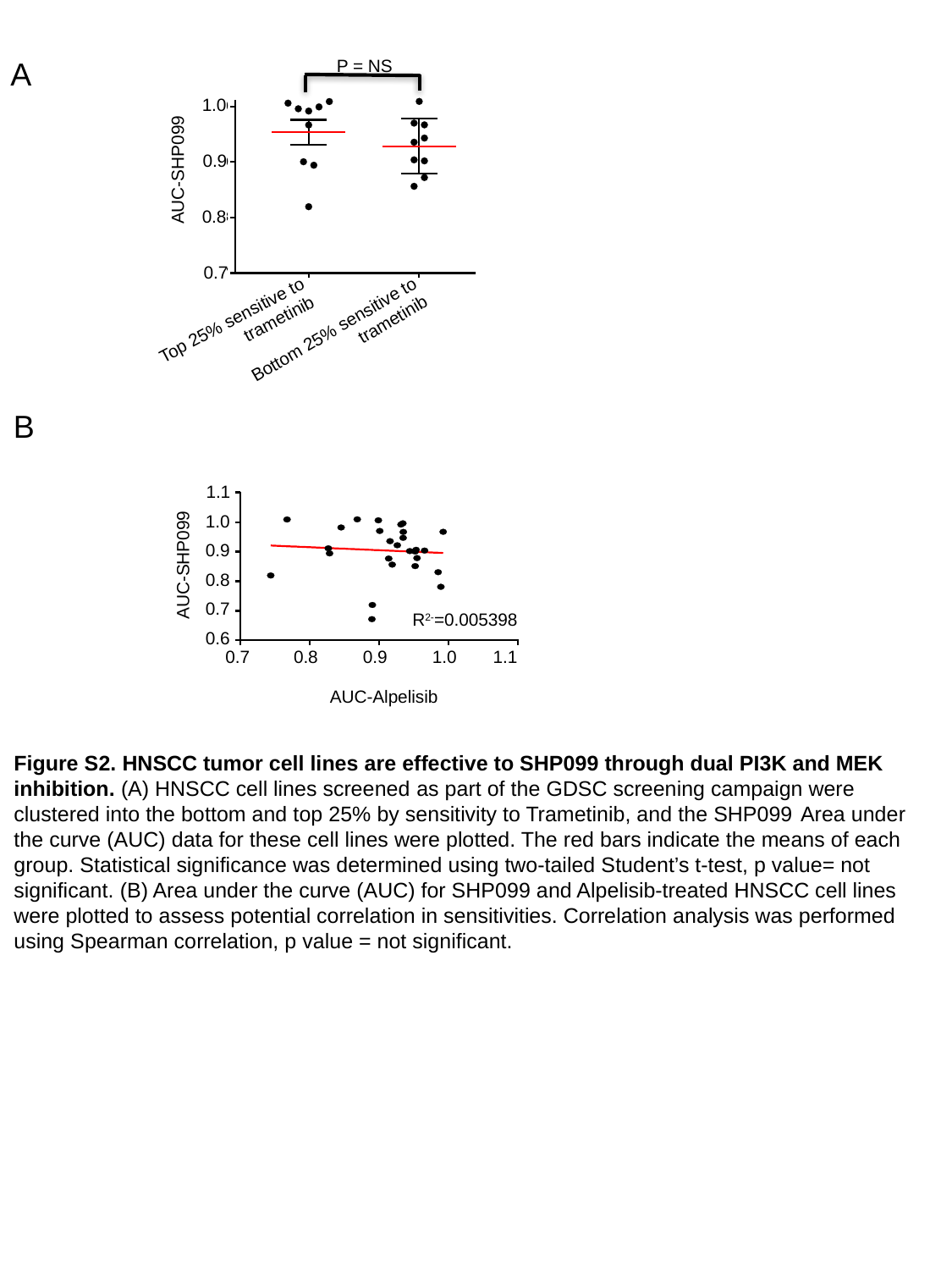

A
P = NS
1.0
0.9
0.8
AUC-SHP099
0.7
Top 25% sensitive to trametinib
Bottom 25% sensitive to trametinib
B
1.1
1.0
0.9
AUC-SHP099
0.8
0.7
R2-=0.005398
0.6
0.7
0.8
0.9
1.0
1.1
AUC-Alpelisib
Figure S2. HNSCC tumor cell lines are effective to SHP099 through dual PI3K and MEK inhibition. (A) HNSCC cell lines screened as part of the GDSC screening campaign were clustered into the bottom and top 25% by sensitivity to Trametinib, and the SHP099 Area under the curve (AUC) data for these cell lines were plotted. The red bars indicate the means of each group. Statistical significance was determined using two-tailed Student’s t-test, p value= not significant. (B) Area under the curve (AUC) for SHP099 and Alpelisib-treated HNSCC cell lines were plotted to assess potential correlation in sensitivities. Correlation analysis was performed using Spearman correlation, p value = not significant.
