# Supplementary material for: Pharmacologic Inhibition of SHP2 Blocks Both PI3K and MEK Signaling in Low-epiregulin HNSCC via GAB1
Source: Cancer Res Commun. 2022 Sep 26;2(9):1061–74. doi: 10.1158/2767-9764.CRC-21-0137 (PMC9728803; doi:10.1158/2767-9764.CRC-21-0137)
Supplement: Figure S3 — Three- and five-day western blot analysis of SHP099-sensitive and - resistant HNSCC cell lines [file crc-21-0137-s03.pptx]

## Slide 1
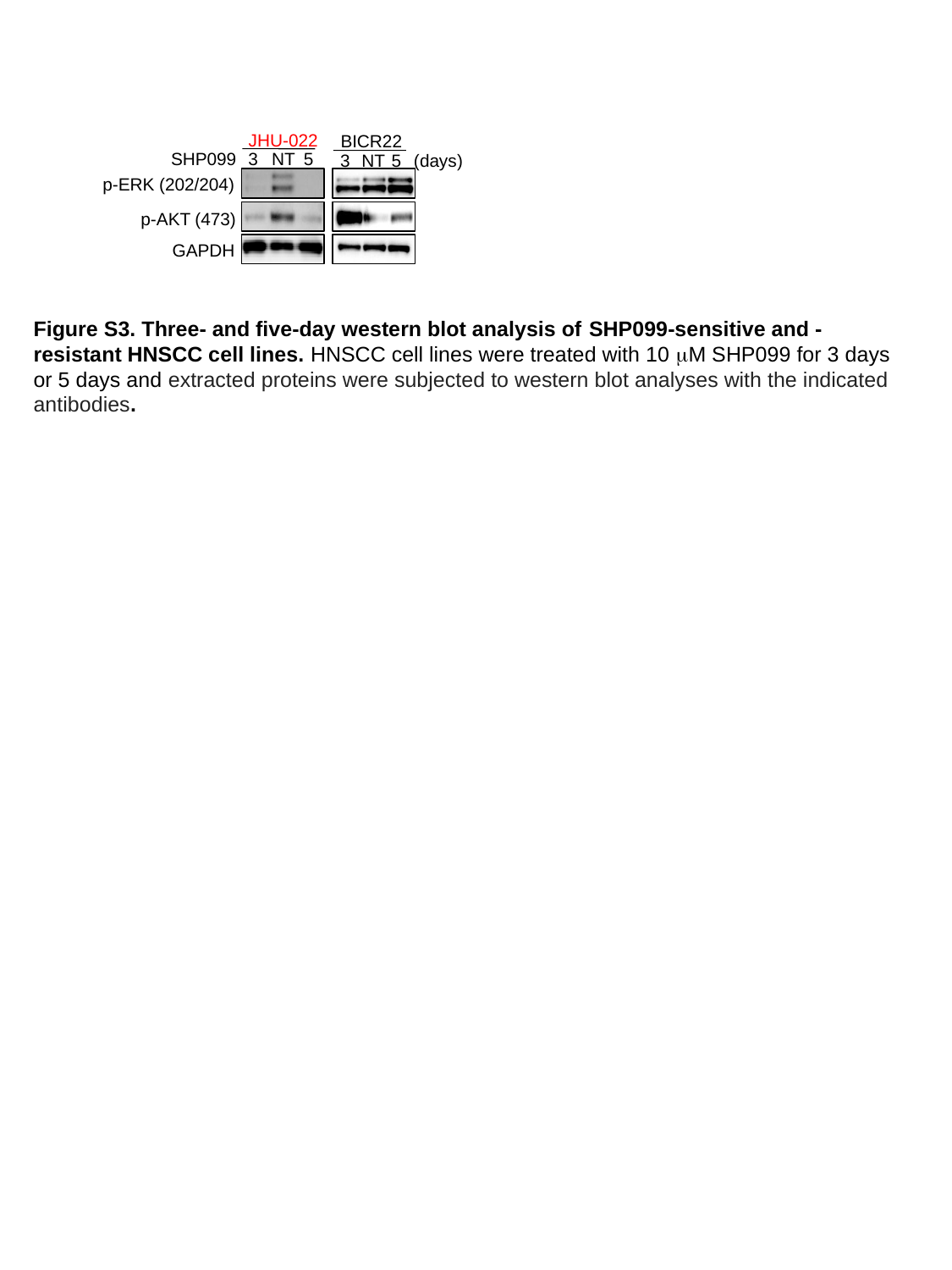

JHU-022
BICR22
SHP099
3
NT
5
3
NT
5
(days)
p-ERK (202/204)
p-AKT (473)
GAPDH
Figure S3. Three- and five-day western blot analysis of SHP099-sensitive and - resistant HNSCC cell lines. HNSCC cell lines were treated with 10 μM SHP099 for 3 days or 5 days and extracted proteins were subjected to western blot analyses with the indicated antibodies.
