# Supplementary material for: Pharmacologic Inhibition of SHP2 Blocks Both PI3K and MEK Signaling in Low-epiregulin HNSCC via GAB1
Source: Cancer Res Commun. 2022 Sep 26;2(9):1061–74. doi: 10.1158/2767-9764.CRC-21-0137 (PMC9728803; doi:10.1158/2767-9764.CRC-21-0137)
Supplement: Figure S4 — The mechanism of action of SHP099 inhibition [file crc-21-0137-s04.pptx]

## Slide 1
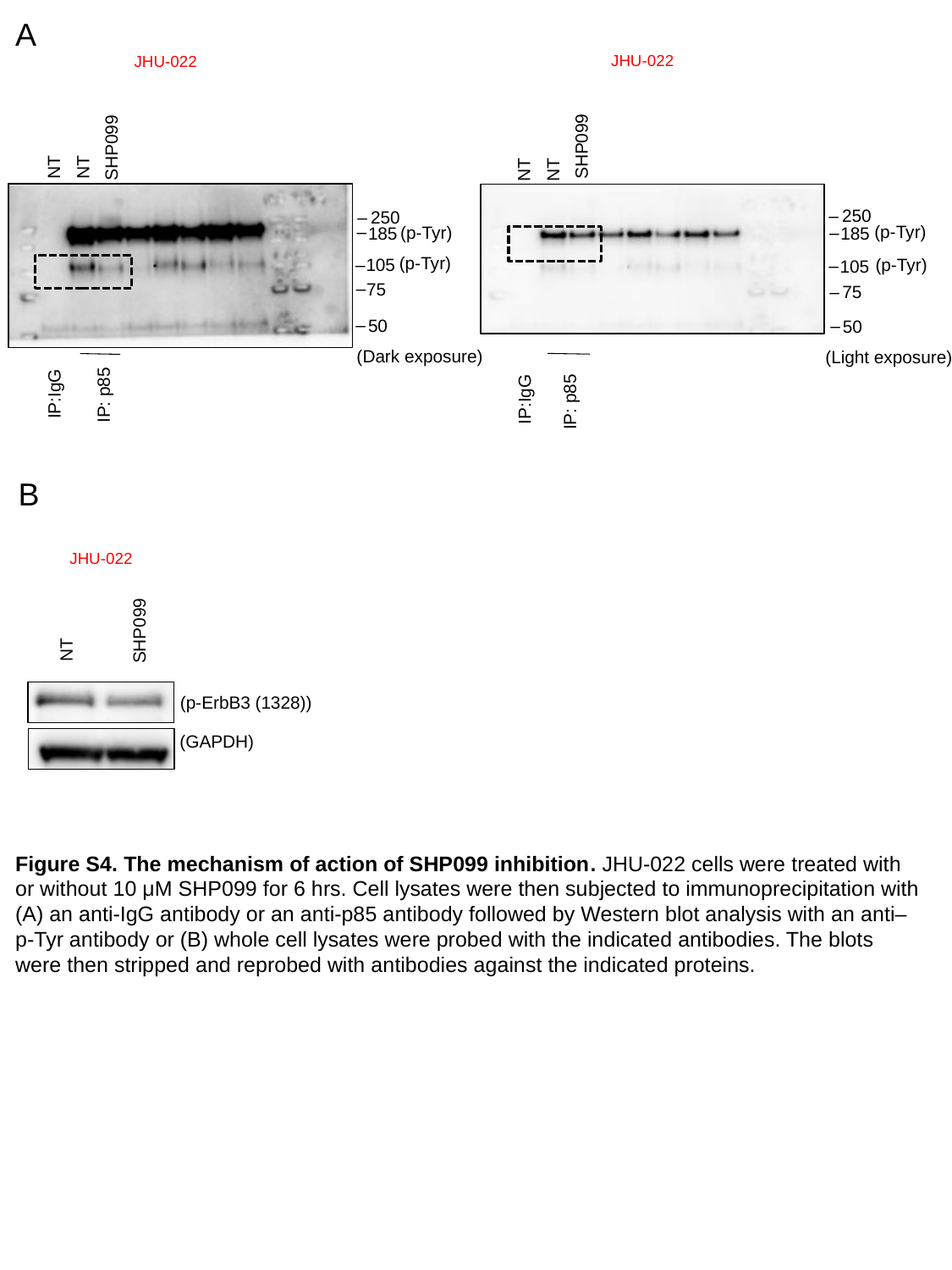

A
JHU-022
JHU-022
SHP099
SHP099
NT
NT
NT
NT
–
250
–
250
 (p-Tyr)
–
–
 (p-Tyr)
185
185
 (p-Tyr)
 (p-Tyr)
–
105
–
105
–
75
–
75
–
50
–
50
 (Dark exposure)
 (Light exposure)
IP:IgG
IP: p85
IP:IgG
IP: p85
B
JHU-022
SHP099
NT
 (p-ErbB3 (1328))
 (GAPDH)
Figure S4. The mechanism of action of SHP099 inhibition. JHU-022 cells were treated with or without 10 μM SHP099 for 6 hrs. Cell lysates were then subjected to immunoprecipitation with (A) an anti-IgG antibody or an anti-p85 antibody followed by Western blot analysis with an anti–p-Tyr antibody or (B) whole cell lysates were probed with the indicated antibodies. The blots were then stripped and reprobed with antibodies against the indicated proteins.
