# Supplementary material for: Pharmacologic Inhibition of SHP2 Blocks Both PI3K and MEK Signaling in Low-epiregulin HNSCC via GAB1
Source: Cancer Res Commun. 2022 Sep 26;2(9):1061–74. doi: 10.1158/2767-9764.CRC-21-0137 (PMC9728803; doi:10.1158/2767-9764.CRC-21-0137)
Supplement: Figure S5 — Western blot analysis of ErbB members [file crc-21-0137-s05.pptx]

## Slide 1
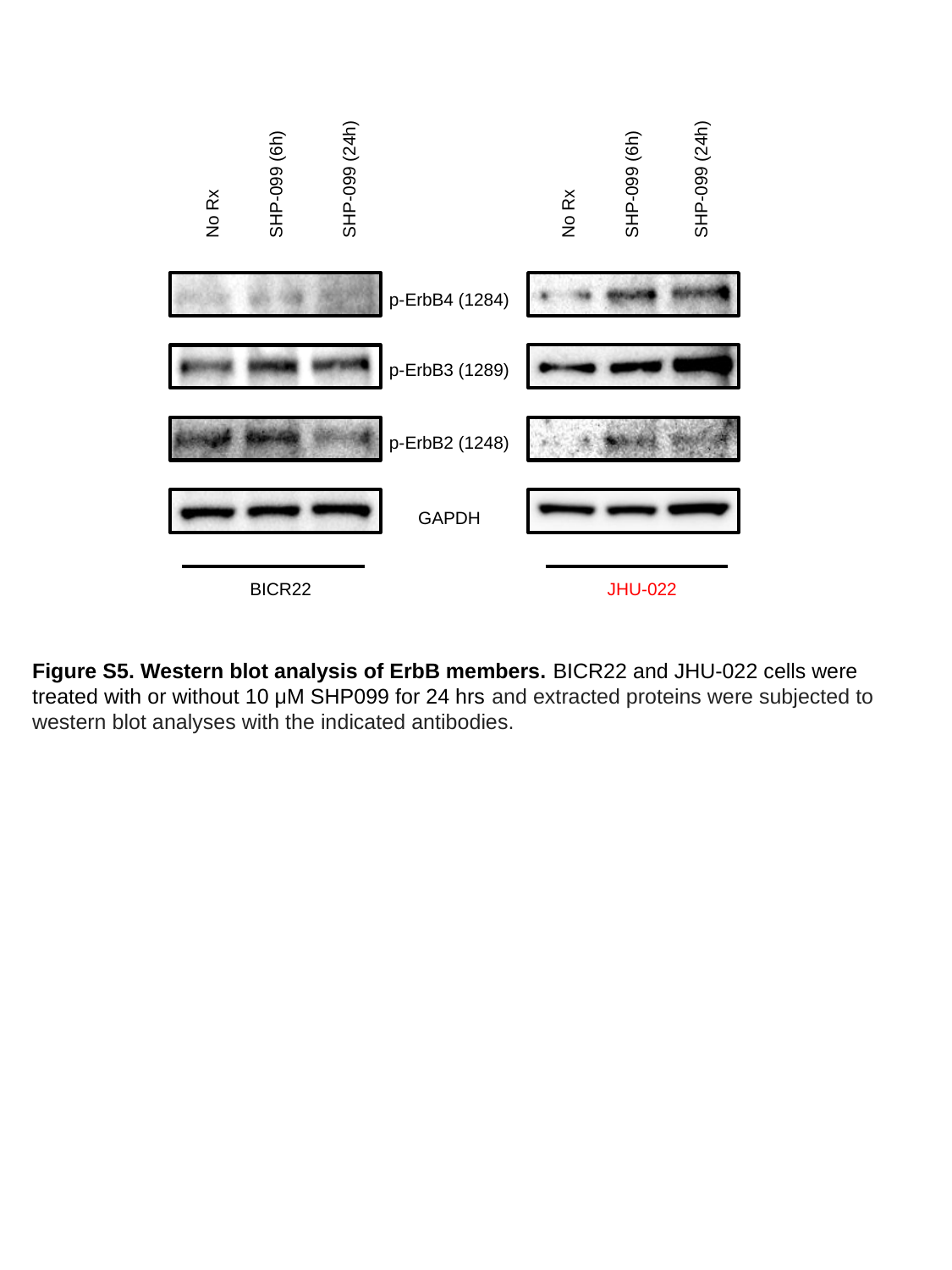

SHP-099 (24h)
SHP-099 (24h)
SHP-099 (6h)
SHP-099 (6h)
No Rx
No Rx
p-ErbB4 (1284)
p-ErbB3 (1289)
p-ErbB2 (1248)
GAPDH
JHU-022
BICR22
Figure S5. Western blot analysis of ErbB members. BICR22 and JHU-022 cells were treated with or without 10 μM SHP099 for 24 hrs and extracted proteins were subjected to western blot analyses with the indicated antibodies.
