# Supplementary material for: Pharmacologic Inhibition of SHP2 Blocks Both PI3K and MEK Signaling in Low-epiregulin HNSCC via GAB1
Source: Cancer Res Commun. 2022 Sep 26;2(9):1061–74. doi: 10.1158/2767-9764.CRC-21-0137 (PMC9728803; doi:10.1158/2767-9764.CRC-21-0137)
Supplement: Figure S6 — Weight profiles of xenograft models treated with SHP099 [file crc-21-0137-s06.pptx]

## Slide 1
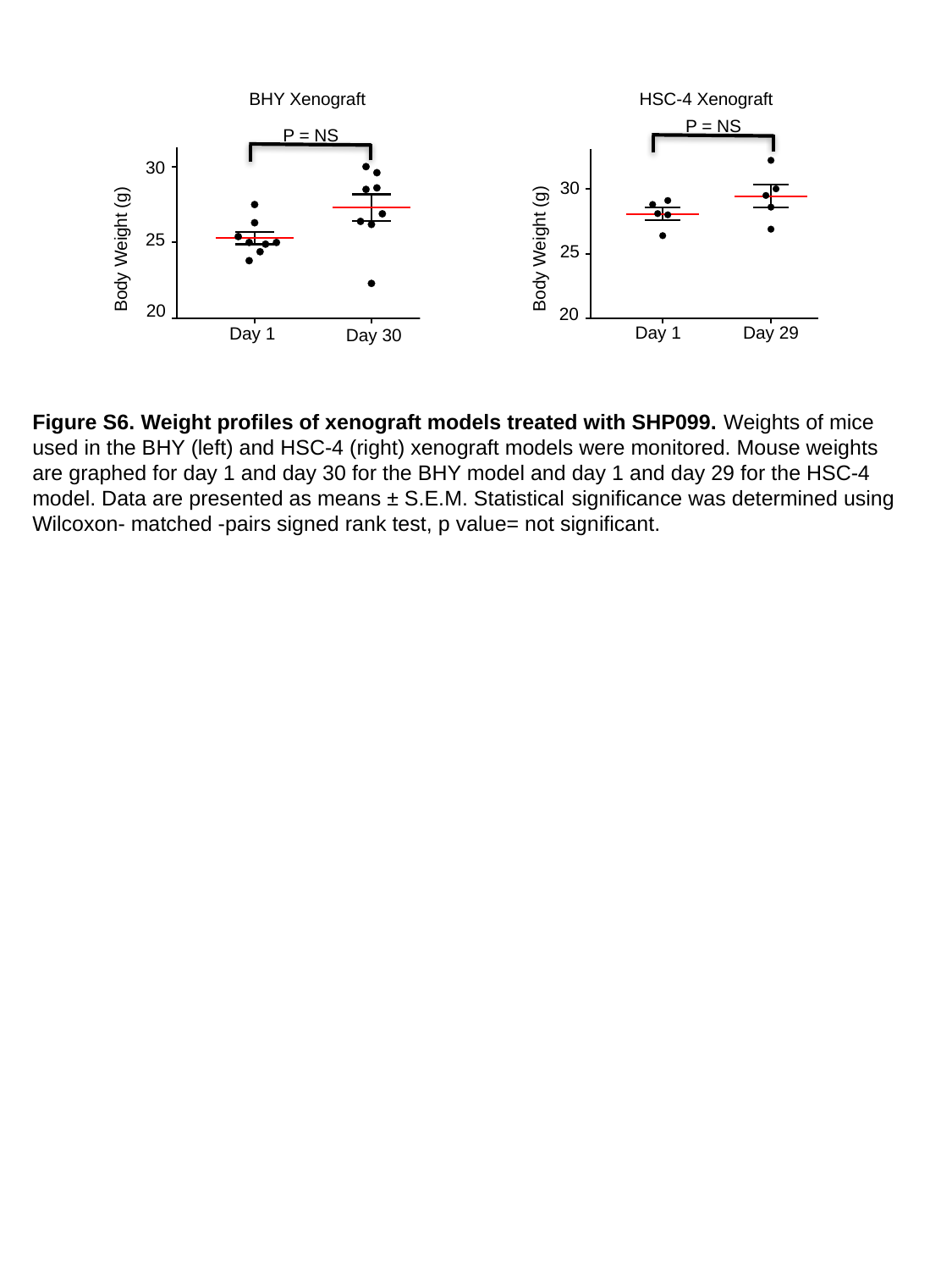

BHY Xenograft
HSC-4 Xenograft
P = NS
P = NS
30
30
Body Weight (g)
Body Weight (g)
25
25
20
20
Day 1
Day 29
Day 1
Day 30
Figure S6. Weight profiles of xenograft models treated with SHP099. Weights of mice used in the BHY (left) and HSC-4 (right) xenograft models were monitored. Mouse weights are graphed for day 1 and day 30 for the BHY model and day 1 and day 29 for the HSC-4 model. Data are presented as means ± S.E.M. Statistical significance was determined using Wilcoxon- matched -pairs signed rank test, p value= not significant.
