# Supplementary material for: Pharmacologic Inhibition of SHP2 Blocks Both PI3K and MEK Signaling in Low-epiregulin HNSCC via GAB1
Source: Cancer Res Commun. 2022 Sep 26;2(9):1061–74. doi: 10.1158/2767-9764.CRC-21-0137 (PMC9728803; doi:10.1158/2767-9764.CRC-21-0137)
Supplement: Figure S7 — RTK ligand expression in HNSCC cell lines [file crc-21-0137-s07.pptx]

## Slide 1
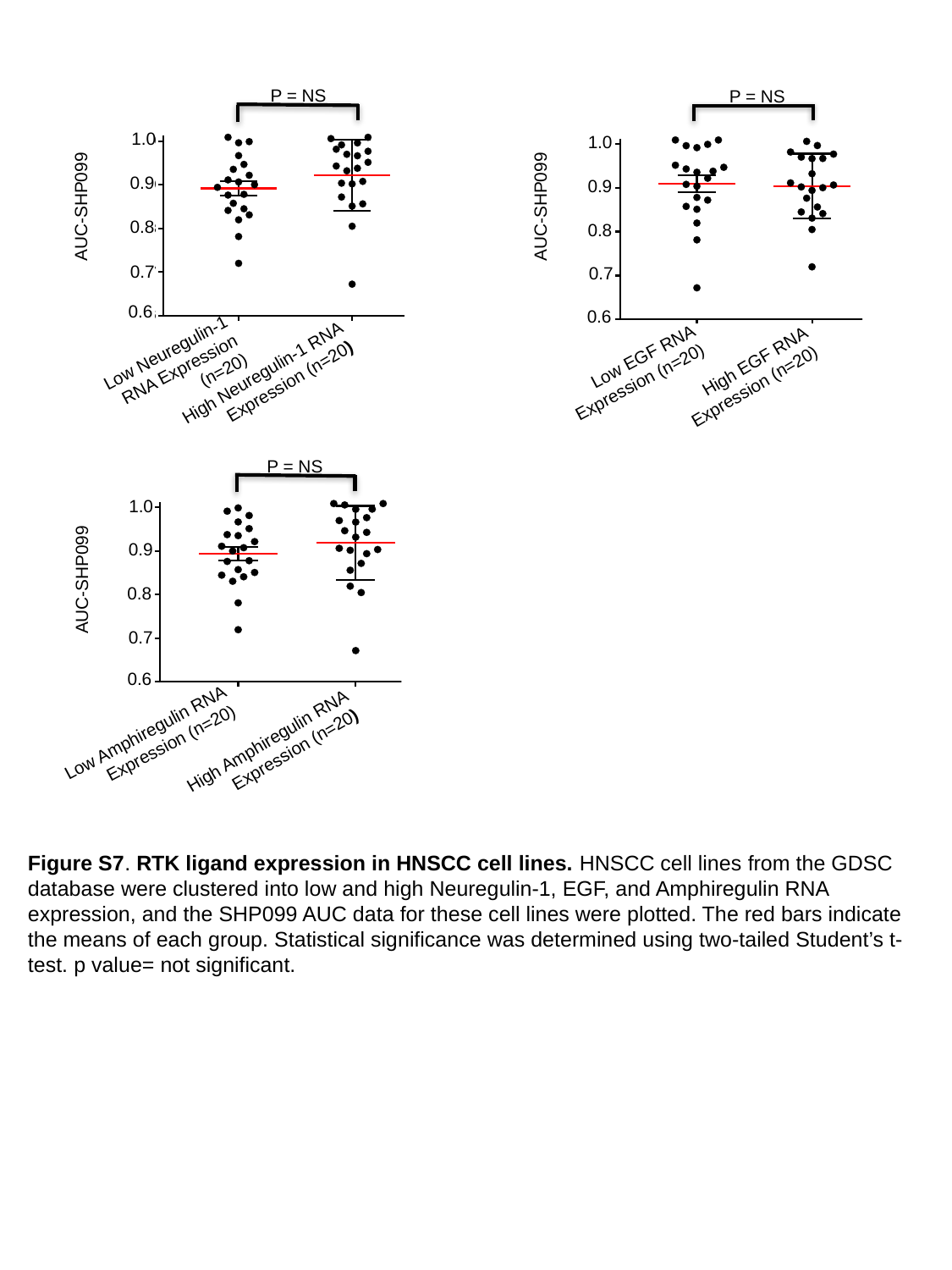

P = NS
P = NS
1.0
1.0
0.9
0.9
0.8
0.8
AUC-SHP099
AUC-SHP099
0.7
0.7
0.6
0.6
Low Neuregulin-1 RNA Expression (n=20)
Low EGF RNA Expression (n=20)
High EGF RNA Expression (n=20)
High Neuregulin-1 RNA Expression (n=20)
P = NS
1.0
0.9
0.8
AUC-SHP099
0.7
0.6
Low Amphiregulin RNA Expression (n=20)
High Amphiregulin RNA Expression (n=20)
Figure S7. RTK ligand expression in HNSCC cell lines. HNSCC cell lines from the GDSC database were clustered into low and high Neuregulin-1, EGF, and Amphiregulin RNA expression, and the SHP099 AUC data for these cell lines were plotted. The red bars indicate the means of each group. Statistical significance was determined using two-tailed Student’s t-test. p value= not significant.
