# Supplementary material for: Pharmacologic Inhibition of SHP2 Blocks Both PI3K and MEK Signaling in Low-epiregulin HNSCC via GAB1
Source: Cancer Res Commun. 2022 Sep 26;2(9):1061–74. doi: 10.1158/2767-9764.CRC-21-0137 (PMC9728803; doi:10.1158/2767-9764.CRC-21-0137)
Supplement: Figure S8 — Neuregulin-1 does not rescue PI3K and MEK signaling in HNSCC treated with SHP2 inhibitor [file crc-21-0137-s08.pptx]

## Slide 1
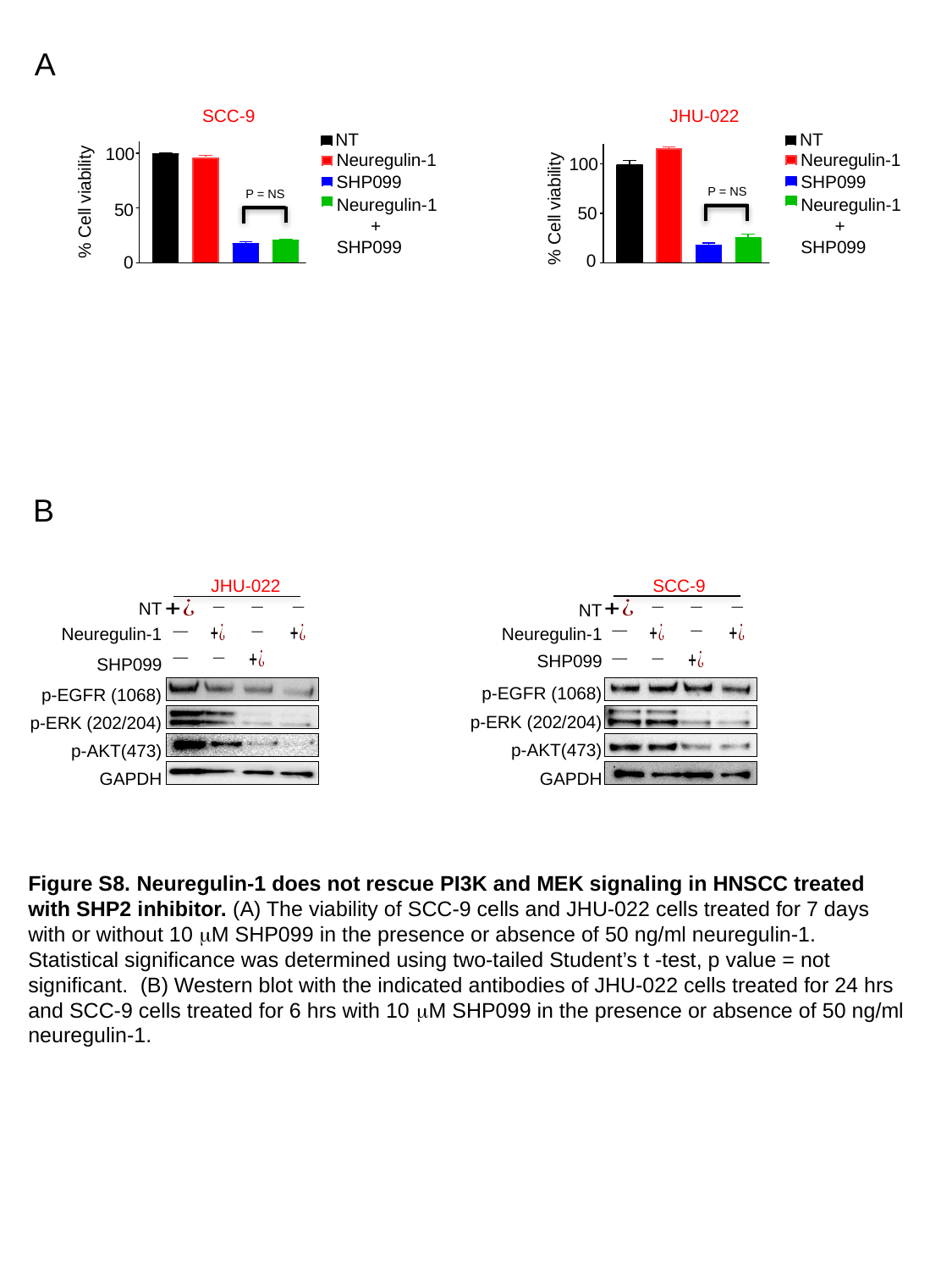

A
SCC-9
NT
100
Neuregulin-1
% Cell viability
SHP099
P = NS
Neuregulin-1
 +
SHP099
50
 0
JHU-022
NT
Neuregulin-1
100
SHP099
P = NS
Neuregulin-1
 +
SHP099
50
 0
% Cell viability
B
SCC-9
NT
Neuregulin-1
SHP099
p-EGFR (1068)
p-ERK (202/204)
p-AKT(473)
GAPDH
JHU-022
NT
Neuregulin-1
SHP099
p-EGFR (1068)
p-ERK (202/204)
p-AKT(473)
GAPDH
Figure S8. Neuregulin-1 does not rescue PI3K and MEK signaling in HNSCC treated with SHP2 inhibitor. (A) The viability of SCC-9 cells and JHU-022 cells treated for 7 days with or without 10 μM SHP099 in the presence or absence of 50 ng/ml neuregulin-1. Statistical significance was determined using two-tailed Student’s t -test, p value = not significant. (B) Western blot with the indicated antibodies of JHU-022 cells treated for 24 hrs and SCC-9 cells treated for 6 hrs with 10 μM SHP099 in the presence or absence of 50 ng/ml neuregulin-1.
