# Supplementary material for: Pharmacologic Inhibition of SHP2 Blocks Both PI3K and MEK Signaling in Low-epiregulin HNSCC via GAB1
Source: Cancer Res Commun. 2022 Sep 26;2(9):1061–74. doi: 10.1158/2767-9764.CRC-21-0137 (PMC9728803; doi:10.1158/2767-9764.CRC-21-0137)
Supplement: Figure S9 — Biological importance of Gab1 in HNSCC cells [file crc-21-0137-s09.pptx]

## Slide 1
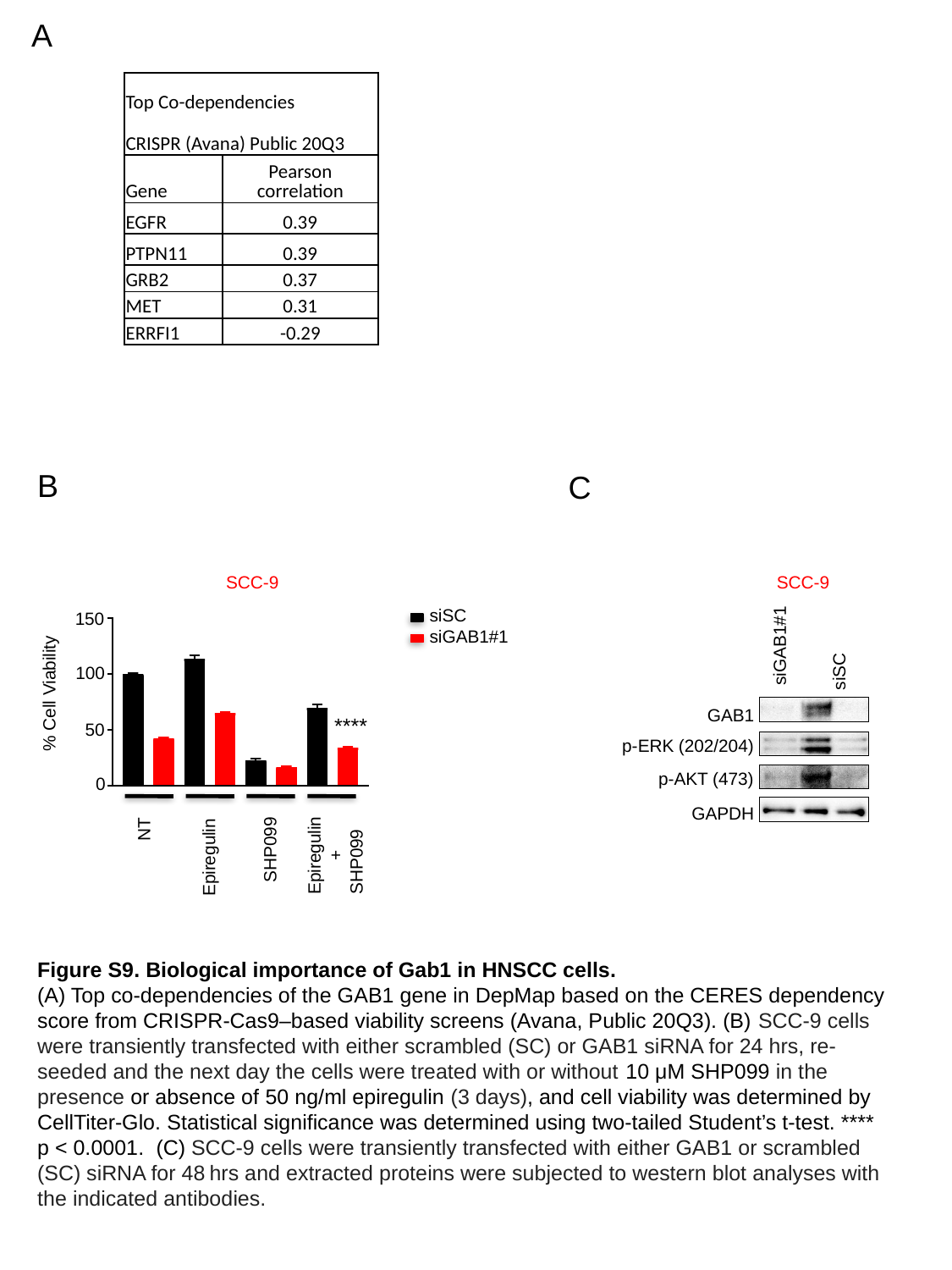

A
| Top Co-dependencies | |
| --- | --- |
| CRISPR (Avana) Public 20Q3 | |
| Gene | Pearson correlation |
| EGFR | 0.39 |
| PTPN11 | 0.39 |
| GRB2 | 0.37 |
| MET | 0.31 |
| ERRFI1 | -0.29 |
B
C
SCC-9
SCC-9
siSC
siGAB1#1
150
siGAB1#1
siSC
100
% Cell Viability
GAB1
****
50
p-ERK (202/204)
p-AKT (473)
 0
GAPDH
Epiregulin
 +
SHP099
NT
SHP099
Epiregulin
Figure S9. Biological importance of Gab1 in HNSCC cells.
(A) Top co-dependencies of the GAB1 gene in DepMap based on the CERES dependency score from CRISPR-Cas9–based viability screens (Avana, Public 20Q3). (B) SCC-9 cells were transiently transfected with either scrambled (SC) or GAB1 siRNA for 24 hrs, re-seeded and the next day the cells were treated with or without 10 μM SHP099 in the presence or absence of 50 ng/ml epiregulin (3 days), and cell viability was determined by CellTiter-Glo. Statistical significance was determined using two-tailed Student’s t-test. **** p < 0.0001. (C) SCC-9 cells were transiently transfected with either GAB1 or scrambled (SC) siRNA for 48 hrs and extracted proteins were subjected to western blot analyses with the indicated antibodies.
