# Supplementary material for: Pharmacologic Inhibition of SHP2 Blocks Both PI3K and MEK Signaling in Low-epiregulin HNSCC via GAB1
Source: Cancer Res Commun. 2022 Sep 26;2(9):1061–74. doi: 10.1158/2767-9764.CRC-21-0137 (PMC9728803; doi:10.1158/2767-9764.CRC-21-0137)
Supplement: Figure S10 — Effects of knockdown of Gab1 in SHP099-resistant HNSCC cell line [file crc-21-0137-s10.pptx]

## Slide 1
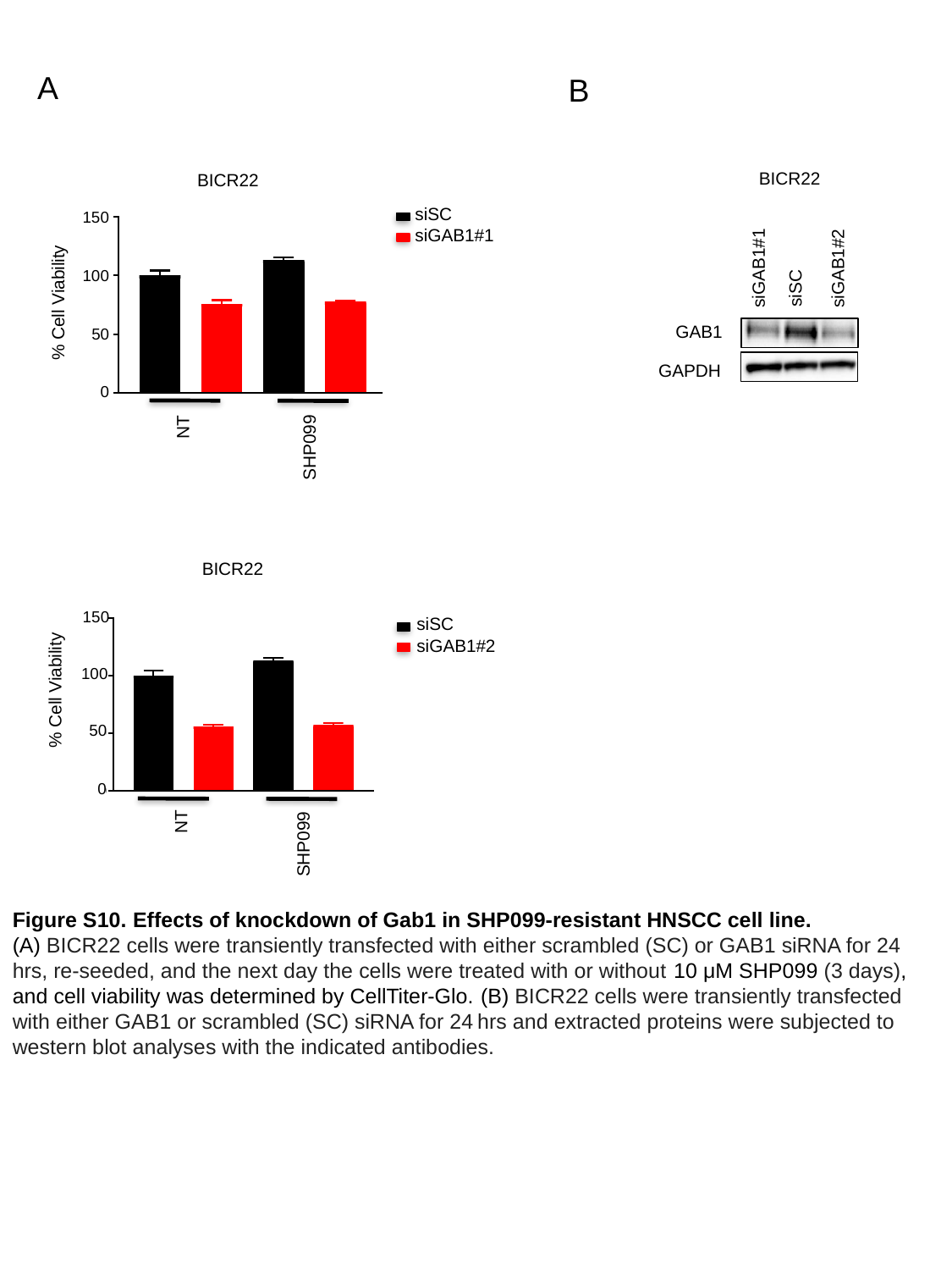

A
B
BICR22
BICR22
siSC
siGAB1#1
150
siGAB1#1
siGAB1#2
100
siSC
% Cell Viability
GAB1
50
GAPDH
0
NT
SHP099
BICR22
150
siSC
siGAB1#2
100
% Cell Viability
50
0
NT
SHP099
Figure S10. Effects of knockdown of Gab1 in SHP099-resistant HNSCC cell line.
(A) BICR22 cells were transiently transfected with either scrambled (SC) or GAB1 siRNA for 24 hrs, re-seeded, and the next day the cells were treated with or without 10 μM SHP099 (3 days), and cell viability was determined by CellTiter-Glo. (B) BICR22 cells were transiently transfected with either GAB1 or scrambled (SC) siRNA for 24 hrs and extracted proteins were subjected to western blot analyses with the indicated antibodies.
